# Unveiling the structural insights and inhibitory potential of coumarin-1,2,3-triazole hybrids against BACE1: a promising approach for Alzheimer’s disease therapy

**DOI:** 10.3389/fchem.2026.1824875

**Published:** 2026-07-07

**Authors:** Mervt Almostafa, Iqra Ali, Galal Yahya, Abdul Rauf Siddiqi

**Affiliations:** 1 Department of Chemistry, College of Science, King Faisal University, Alhofuf, Saudi Arabia; 2 Department of Biosciences, COMSATS University Islamabad, Islamabad Campus, Islamabad, Pakistan; 3 Department of Microbiology and Immunology, Faculty of Pharmacy, Zagazig University, Al Sharqia, Egypt; 4 Molecular Biology Institute of Barcelona (IBMB), CSIC, Barcelona, Spain

**Keywords:** ADMET analysis, alzheimer’s disease, amyloid beta (aβ), BACE1 (β-secretase), coumarin-1,2,3-triazole hybrids, molecular dynamics simulation

## Abstract

Alzheimer’s disease (AD) is a progressive neurodegenerative disorder characterized by amyloid beta (Aβ) plaque accumulation, in which β-secretase (BACE1) plays a central role in the amyloidogenic pathway. Inhibition of BACE1 represents a promising therapeutic strategy; however, effective and safe inhibitors remain limited. This study aimed to identify novel coumarin-1,2,3-triazole hybrids as potential BACE1 inhibitors using comprehensive *in silico* approaches. Molecular shape and distance-based features derived from co-crystal ligands were used to generate and validate a pharmacophore model through ROC-based area under the curve analysis. A designed library of hybrids underwent pharmacophore-based virtual screening, yielding 92 hits with ≤0.6 RMSD. These compounds were further evaluated via structure-based molecular docking, from which six top candidates were selected, demonstrating superior docking scores relative to reported inhibitors. ADMET and density functional theory analyses indicated favorable pharmacokinetic profiles and electronic properties, highlighting potential reactive sites. The selected compound, CUM-0199, was subsequently validated through a 200 ns molecular dynamics simulation and MMGBSA binding free energy analysis, confirming structural stability and favourable binding affinity toward BACE1. Overall, the findings suggest CUM-0199 as a potential lead candidate for AD therapy, warranting further experimental validation and clinical investigation.

## Graphical Abstract

**Figure:**
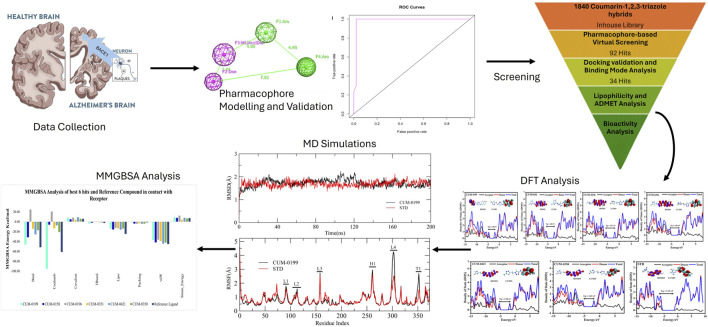

## 1 Introduction

Alzheimer’s disease (AD) is a debilitating and progressive neurodegenerative brain disorder that damages the brain and impairs cognitive functions, particularly memory. The symptoms of AD deteriorate over time, ultimately leading to the loss of independence and the requirement for full-time care (Abubakar et al., 2022). It is the major cause of dementia in the elderly, and its prevalence is increasing globally. The incidence of AD has a significant influence on the lives of the patient’s family, as well as a considerable financial cost to society (Abreu et al., 2005). The World Alzheimer’s Report 2021 estimates that 55 M people are living with Alzheimer’s disease and others with dementia (Gauthier et al., 2021). The number is expected to rise to 152 M by the year 2050 (Li et al., 2022). It’s the sixth most common cause of mortality in the US and the fifth most common worldwide (Alzheimer’s Association, 2025).

The pathogenesis of AD is complex, and the precise etiology of Alzheimer’s disease is unclear. It is considered to be caused by a complex interplay of genetic, environmental, and lifestyle factors (Barnes and Yaffe, 2011) along with inflammation, neurodegeneration, and neurofibrillary tangles (NFTs) in the brain. Key contributors to AD include BACE1, which cleaves amyloid precursor protein at the β-site within the extracellular domain, and the accumulation of two abnormal proteins, i.e., amyloid beta (Aβ) and hyperphosphorylated tau proteins (Silva et al., 2019) that interfere with the communication between nerve cells, leading to cell death and brain shrinkage (Selkoe, 2001). As the disease advances, affected individuals experience cognitive decline, memory loss, and behavioral changes. Some other variables, i.e., oxidative stress, lead to the formation of senile plaques and accumulation of NFTs (Huang, Zhang, and Chen, 2016). Furthermore, increasing levels of inflammatory cytokines and related genes have been linked to the development of AD (Hollingworth et al., 2011). Mutations in genes such as amyloid precursor protein (APP), presenilin-1 (PSEN1), and presenilin-2 (PSEN2) have been identified as associated with the onset of familial Alzheimer’s disease, a rare form of the ailment that affects individuals with a family history of the condition (Guerreiro and Hardy, 2014). Additionally, other identified risk factors encompass a history of head injury, elevated blood pressure, and increased cholesterol levels (Sperling et al., 2011).

Beta-secretase 1 (BACE1), a transmembrane aspartyl protease enzyme, is involved in the formation of Aβ peptides, which are one of the medical hallmarks of AD. To reduce the formation of amyloid beta peptides in AD patients, it’s necessary to inhibit BACE1 activity, as it is one of the key contributors involved in amyloidogenic processing. BACE1 is a challenging central nervous system (CNS) drug target. Understanding the structure and function of BACE1 provides valuable insights for designing potential inhibitors as drug candidates for the treatment of AD.

There is presently no proper cure for Alzheimer’s disease, but there are medications available that provide temporary symptomatic relief in some patients, but do not cure or halt the progression of AD, including cholinesterase inhibitors, memantine, N-methyl-D-aspartate (NMDA) receptor antagonists, and TRx0237, also known as HMTM (Hydromethylthionine mesylate) (Congdon and Sigurdsson, 2018). However, the results of some clinical trials have been inconsistent, while some have negative effects on cognitive functions, and some have been rejected by the FDA due to conflicting data in clinical trials (Imai et al., 2020). Clinical success of BACE1 inhibitors has been limited by safety concerns and a lack of significant cognitive benefits. Moreover, some Aβ-targeting vaccines reduce mild amyloid burden in Alzheimer’s disease, including CAD106, ACI-24, and UB-311. None of these medications have yet received regulatory approval to treat AD, as they are still in the evaluation phase (Rafii et al., 2022; Winblad et al., 2012; Yu et al., 2023). Non-pharmacological interventions, such as cognitive training, physical activity, and social interaction, have also been found to improve cognitive function and quality of life in individuals with AD (Clare et al., 2003).

As the global population ages, the incidence of AD is expected to rise. It is therefore important for researchers to continue studying such diseases and developing new treatments and interventions that can improve the standard of living for individuals with AD and their families. Several therapeutic approaches are currently being investigated, but further research is needed to develop effective treatments for this devastating disease (Prince et al., 2013). Most importantly, innovative therapeutic scaffolds with minimal side effects remain an urgent priority for medicinal chemists. The purpose of this study is to determine whether Coumarine-1,2,3-triazole hybrids with neuroprotective effects can inhibit BACE1 and help overcome AD.

The coumarin-1,2,3-triazole hybrids belong to a distinct class of benzopyrone compounds, which gained attention as potential candidates for their neuroprotective, anti-inflammatory, and anti-amyloidogenic properties (Lin et al., 2022). These synthetic compounds were designed to integrate structural features associated with both coumarin and 1,2,3-triazole moieties in order to increase their biological activity, stability, bioavailability, and therapeutic potential (Fan et al., 2018). They have been the subject of extensive research due to their neuroprotective effects (Flores-Morales et al., 2023; Khan et al., 2023), which are essential in combating neurodegenerative processes associated with AD. Moreover, some studies reported their effects as anti-diabetic, antioxidant, anti-tumor, antimicrobial, and antibacterial properties (Basappa et al., 2020; Kahveci et al., 2017; Shaikh et al., 2025). Reducing inflammation and inhibiting amyloid-beta aggregation are key strategies for addressing the underlying pathologies of the disease.

## 2 Materials and methods

**FIGURE 1:**
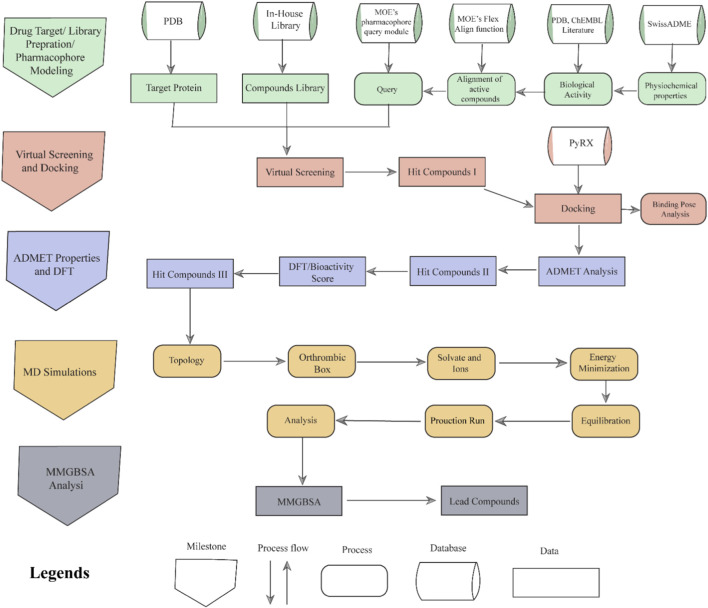
Flowchart of proposed study.

The general flowchart of the methodology employed in the research project is depicted in Figure 1. Legends were given at the bottom of the flowchart to understand the proposed study.

### 2.1 Pharmacophore modelling and validation

40 cocrystal structures of BACE1 were retrieved from the RCS protein data bank (https://www.rcsb.org/), while their activity was obtained from ChEMBL (https://www.ebi.ac.uk/chembl/) database and literature review. Physicochemical properties of all cocrystal compounds were determined via the SwissADME (http://www.swissadme.ch/index.php) webserver. Poses and interaction information of cocrystal complexes were explored. The active ligands and their conformations were aligned via the flex align module to extract common shape and distance-based features that play a significant role in biological activity (Qing et al., 2014). Stochastic conformational search along iteration limit 20, failure limit 10, and energy cutoff value of 10 was set for alignment purposes. C27 depicts the key interaction pattern across the majority of co-crystalized ligands. Therefore, the co-crystal ligand with the highest activity was selected. The pharmacophore query was generated on template compound C27, which is a representative of all superimposed ligands. Common key features were added to generate a pharmacophore query with a 1.2 tolerance and 50% threshold value. Before virtual screening, the pharmacophore model was validated to assess its capability and specificity in discriminating between active and inactive compounds from a dataset comprising both active and decoy compounds. For this, a prepared dataset of 80 active compounds from the PDB and ChEMBL databases was used, based on their structural and physicochemical properties. Moreover, a library of 285 decoys from a Database of Useful Decoys Enhanced (DUD.E) was selected, which used similar physicochemical properties of C27, along with dissimilar 2D topology.

### 2.2 Library designing and virtual screening

An in-house library of 1840 coumarin-1,2,3-triazole hybrids was prepared by substitution of distinct functional groups at the 12th position to get potential compounds targeting beta-secretase 1. Coumarin is well known for its anti-inflammatory and neuroprotective properties, which are significant for the treatment of AD (Hu et al., 2019). 1,2,3-triazoles are employed in medicinal chemistry as useful building blocks due to their stability, simplicity in synthesis, and potential for diverse interactions with distinct biological targets. When coupled with coumarin, they can display elevated pharmacological properties and target-specific interactions, making them attractive candidates for AD therapy (Bozorov, Zhao, and Aisa, 2019). Designed compounds depict good synthetic accessibility scores and fulfill the RO5 (Lipinski rule of 5) criteria. The pharmacophore model was employed to screen 1840 coumarin-1,2,3-triazole hybrids and resultantly get potential hits.

### 2.3 Structure-based molecular docking

Docking-based screening was performed to further screen hits and to identify potential ligands that depict a high probability of binding with beta-secretase 1. The macromolecule was preprocessed by eliminating water molecules, ions, and ligands (Ali, Rasheed, et al., 2023). Furthermore, fix the side chain atoms of residues. Then, polar hydrogens and charges were added to ensure that the receptor is properly prepared to capture important molecular interactions and accurately predict preferred binding modes. Coumarin-1,2,3-triazole hybrids were optimized and minimized by employing the UFF force field along the conjugate gradient optimization algorithm over 200 iterations. However, the energy difference was less than 0.1, and then the files were converted into pdbqt format. For blind docking, the size of grid coordinates was X = 64, Y = 76, Z = 57, which covers the whole protein. Blind docking helps to determine whether the hit compounds bind to the active site, other binding sites, or allosteric sites. Prepared receptor and ligands were subjected to molecular docking via PyRx 0.8. x software (Dallakyan and Olson, 2014). The software’s protocol was validated via redocking and cross-docking analysis, demonstrating its robustness and liability. Moreover, 2D and 3D depictions were subjected to Discovery Studio and PyMOL, respectively. The binding poses were analyzed by considering factors such as binding scores, RMSD values between native and docked poses, as well as distances among ligands and binding residues of BACE1.

### 2.4 ADMET analysis of top hits

Physicochemical properties, pharmacokinetic features (i.e., absorption, distribution, metabolism, excretion), and safety profile of potential hits were studied via SwissADME (Daina, Michielin, and Zoete, 2017), ADMETlab 3.0 (Fu et al., 2024), and Osiris Property Explorer, which employs integrated machine learning and deep learning models trained on experimental datasets. ADME screening predicts how effectively the drug will be administered by the body in its active form. How it will be distributed with the ability to cross biological barriers, such as the blood-brain barrier, and reach target tissues. How rapidly it will be metabolized and excreted from the body, with special focus on renal, biliary, and fecal excretion. Toxicity testing predicts the potential adverse effects and safety issues associated with selected compounds. ADMET analysis provides critical data for assessing safety, minimizing the likelihood of late-stage drug failure, and guaranteeing patient safety. Multiple rules and scoring systems have been employed to assess the viability of chemical compounds for further exploration, with an emphasis on factors such as synthetic accessibility, logP values, physicochemical features, and bioavailability.

### 2.5 Bioactivity score prediction

To access the biological activity and efficacy of hit compounds, the PASS online webserver (https://way2drug.com/PassOnline/index.php) was utilized. Top hits against human receptors, i.e., GPCRs, ion channel modulators, numerous kinases, proteases, nuclear receptors, and enzyme inhibitors were explored. Bioactivity scores help to prioritize and predict potential off-target effects. Additionally, these scores assist in observing contrary interactions with unintended biological targets and resultantly minimize the chances of unexpected adverse effects (Ali et al., 2024).

### 2.6 DFT study of coumarin-1,2,3-triazole hybrids

Density functional theory (DFT) and time-dependent density functional theory (TD-DFT) analysis were used to discover the best stable theoretical configuration for the compounds under study. The absence of imaginary frequencies served as an indication for these computational methods that led to the discovery of the energetically ideal state. To do this, optimal computational configurations were used to perform optimization at the ground-state level. Electrostatic potential (ESP) mapping and Density of State (DOS) analyses were also performed, in addition to the standard studies of Frontier molecular orbitals (FMOs), inhibitor characteristics, global chemical reactivity descriptors, and so on (Ali and Abbas, 2022). These elements helped to differentiate the system’s different contributions. Finding the characteristics and findings of the hybrid functional, the Becke-3 exchange functional method, provided by Lee, Yang, Parr (B3LYP), was employed (Russo, Martin, and Hay, 1994) with the basis set 6-31G** required the use of the DFT Package Gaussian 16, Revision C. 01 (Frisch et al., 2016), a software package in conjunction with the GaussView 6.0 program to visualize the findings. All the global reactivity parameters for the studied compounds were computed by utilizing Equations 1-5, where *η*, µ, S, χ,and ω represent hardness, chemical potential, Softness, electronegativity, and electrophilicity index.

$$\eta =\frac{E_{\text{LUMO}\;}+E_{\text{HOMO}\;}}{2}$$ (1)

$$\mu =\frac{E_{\text{LUMO}\;}+E_{\text{HOMO}\;}}{2}$$ (2)

$$S=\frac{1}{\eta }$$ (3)

χ=−μ (4)

$$\omega =\frac{\mu^{2}}{2\eta }$$ (5)

The highest occupied molecular orbital (HOMO), the lowest unoccupied molecular orbital (LUMO), and the HOMO-LUMO energy gaps (E.g.,) are used to describe electronic energy levels of molecules. These energy levels play a crucial role in understanding the interaction between drugs and their target molecules, as well as in predicting various properties of the potential drug molecules themselves. The energy difference between HOMO and LUMO can provide insights into the chemical reactivity of a molecule. Molecules with smaller gaps are generally more reactive because electrons can be more easily excited from the HOMO to the LUMO, facilitating chemical reactions (Abbas et al., 2023a). The HOMO and LUMO orbitals are important variables that show how excited simulated compounds are. The energy gap (E.g.,) is a good way to figure out how chemically reactive the compounds being studied are. The HOMO level acts as the source of electrons, whereas the LUMO level serves as the less effective electron acceptor. Due to the significant energy gap between them, the (HOMO-LUMO) interaction has an adverse impact, leading to the limited efficiency of charge transfer for the inhibitor.

### 2.7 MD simulation study

MD simulations were carried out to understand ligand-receptor binding mechanisms at the atomic level, along with their conformational alterations, utilizing the Desmond software (Bowers et al., 2006). The best binding poses of lead and reference compound were utilized as a starting conformation to run MD simulations. Both studied complexes were preprocessed via Maestro’s protein preparation wizard. Both complexes were minimized to remove steric clashes and unfavorable contacts. A 10Å orthorhombic box of TIP3P was utilized as a solvent model. Complexes further neutralized by the addition of 0.15 M Na^+^ and Cl^−^ ions with the OPLS 2005 force field (Rasheed et al., 2021). The system builder tool was employed to prepare both systems and parameterize them for accurate and efficient molecular dynamics simulations. Both systems employed NPT (constant Number of particles, Pressure, and Temperature) ensemble to stabilize the system at 300 K temperature and 01 atmospheric pressure using Nosé-Hoover chain thermostat. After equilibrium, each MD trajectory was followed over 200 ns. The receptor-ligand interactions were investigated through the simulation interaction diagram tool. MD simulations for both systems were effectively performed on an Intel Core i9-12900 KF processor, equipped with 128 GB RAM, and an MSI GeForce RTX 3080 Ti GPU on a specially built liquid-cooled PC, installed Ubuntu 22.04.1 LTS.

### 2.8 Binding free energy calculation

The binding free energy (ΔG_bind_) for both systems was calculated via prime MM-GBSA to estimate the strength and stability of the studied complexes (Ali et al., 2025). To calculate ΔG_bind_, complexes were optimized and minimized. Counter ions and solvent molecules were added. Default parameters, i.e., OPLS3 force field, dielectric constant along the VSGB solvent model, were applied to compute ΔG_bind_ for both systems. The MD trajectory frames were selected at intervals of 10 ns after the simulation run. ΔG_bind,_ G_complex,_ G_protein,_ G_ligand_ depict total binding free energy, binding free energy of complex, receptor, and ligand, respectively (Equation 6). ΔG_bind_ is the combination of energy in gas and solution phase (Equation 7) (Ali, Iqbal, et al., 2023; Kumar, Shukla, et al., 2023). Gas phase energy (ΔE_gas_) is the combination of van-der Waals, electrostatic, and internal energy (Equation 8). Solvation free energy (ΔGs_ol_) is decomposed into polar (generalized born model) and nonpolar solvation free energy (solvent accessible surface area (SASA)), illustrated in (Equation 9) (Jawad et al., 2019). TΔS denotes conformational entropy expressed in (Equation 10), which was neglected due to high computational cost.

$${\Delta G}_{\text{bind}}{=G}_{\text{complex}}-\left(G_{\text{protein}}{+G}_{\text{ligand}}\right)$$ (6)

$${\Delta G}_{\text{bind}}{=\Delta E}_{\text{gas}}+\Delta Gs_{\text{ol}}-T\Delta S$$ (7)

$${\Delta E}_{\text{gas}}{=\Delta E}_{\mathrm{int}}{+\Delta E}_{\text{ELE}}+\Delta E_{VDW}$$ (8)

$$\Delta Gs_{\text{ol}}{=\Delta G}_{\text{polar}}{+\Delta G}_{\text{nonpolar}}{=\Delta G}_{\text{GB}}+\Delta G_{S}$$ (9)

$$T\Delta S=T\left(\Delta S_{\text{trans}}{+\Delta S}_{\text{rot}}{+\Delta S}_{\text{vib}}\right)$$ (10)

## 3 Results and discussion

### 3.1 Pharmacophore query generation and model quality assessment

**FIGURE 2:**
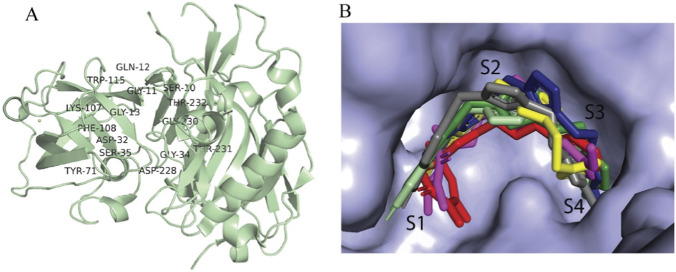
**(A)** Labeled active site residues of the target protein **(B)** Alignment of active compounds in BACE1’s active site.

High-resolution co-crystalized beta-secretase one structures were aligned and analyzed to investigate diverse structural features in its binding site along non-covalent interactions. The best 40 co-crystal structures of active compounds, along with their physicochemical properties and IC_50_ values, were displayed in Supplementary Table S1. Conserved residues of BACE1’s active site were labelled in Figure 2A, while aligned active compounds lay in the sub-pockets S1, S2, S3, and S4 of BACE1 (Figure 2B).

**FIGURE 3:**
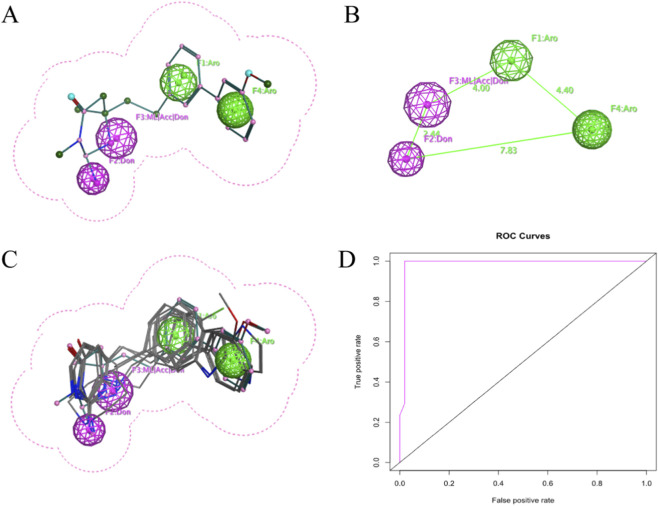
**(A)** Pharmacophore hypothesis with selected features in colored balls and labelled, i.e., green color for aromatic features, while purple for acceptor and donor **(B)** Distance among selected features **(C)**, top aligned co-crystal compounds with selected pharmacophoric features **(D)**, ROC graph for pharmacophore model where false positive represents fraction of decoys and true positive represents fraction of actives. The diagonal black line indicates a random classifier, while the magenta color line represents perfect discrimination and suggests that the model performs very well in classifying compounds correctly.

Based on biological activity, binding poses, common structural and shape-based features, C27 was selected as a reference compound to build a pharmacophore model. The final pharmacophore model contains four pharmacophoric features. The query features were analyzed based on four defined sub-pockets of the active site of BACE1, as displayed in Figure 2B. Both hydrogen bond donor and acceptor lie in the S1 sub-pocket, F1 aromatic feature lies near S2 subsite, while the aromatic ring, the F4 feature, lies near S3 and S4 sub-pockets of BACE1’s active site. Figure 3 depicts the template compound of highest activity along selected features, the distance among selected features, and the area under the ROC curve. The selected pharmacophoric features were F1: second ring (Aro), F2: N24 (Don), F3: N25 (Don/Acc), F4: first ring (Aro) with 100% score and 0.50 radius (Figure 3A). The distance between all selected features was less than 8Å (Figure 3B). Figure 3C displays top-aligned compounds with selected features. Further, the generated pharmacophore query was validated through a receiver operating characteristic (ROC) curve (Figure 3D). The best selected model with an area under the curve (AUC) value of 0.985 has excellent discriminatory power to differentiate active and decoys. Enrichment Factor (EF) for the top 1% was 14.7, while for the top 20% was 4.167, depicting that the pharmacophore model successfully identifies a high proportion of actives compared to random selection, which seems beneficial for virtual screening.

### 3.2 Pharmacophore-based virtual screening

Further, the generated query was employed to screen a prepared in-house library of 1840 coumarin-1,2,3-triazole hybrids and resultantly get 92 hits with an RMSD value of ≤0.6 Å and ≤350 D molecular weight (MW). Generally, lower RMSD values are associated with high structural consistency and stable conformations within the active site (Trott and Olson, 2010), while a stricter MW criterion was used to retain chemical space for subsequent optimization. Hits were carried out for molecular docking to find plausible binding modes. 92 hits along their SMILES, RMSD scores, molecular weight, TPSA, H.B.A., H.B.D., and Lipinski rule of five are shown in Supplementary Table S2.

### 3.3 Validation of docking protocol

**FIGURE 4:**
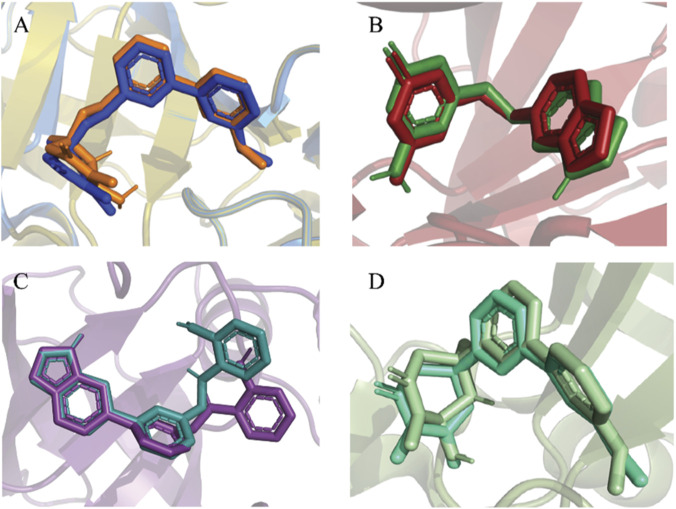
**(A)** redocked pose of ligand with PDB ID: 2VA7 is shown in blue sticks and orange one is the native pose **(B)** redocked pose of ligand with PDB ID: 2VA5 is shown in forest green sticks **(C)** redocked pose of ligand with PDB ID: 2OHT is shown in purple sticks **(D)** redocked pose of ligand with PDB ID: 2VA6 is shown in pale green sticks. The RMSD of redocked poses was less than 1 Å.

Before docking, validate the reliability and accuracy of the PyRx docking protocol via two methods. Initially, redock co-crystal compounds (obtained from co-crystal structures: 2OF0, 2OHQ, 2VA7, 2OHT) at the same binding site, then investigate their binding modes and compare RMSD values. Align all four redocked co-crystal ligands with their native poses, and they depict <1.0 Å RMSD values (Supplementary Table S3), which illustrate the accuracy of docking methodology (Figure 4).

Furthermore, cross-docking analysis was performed as an additional validation approach to check the robustness of the docking protocol. For this, 03 BACE1 complexes were utilized, i.e., 2VA7, 2VA6, 2VA5. Each cocrystal ligand was docked with a non-native protein conformation. Then, calculate the RMSD values for the best-selected poses based on binding affinity and interaction profile, and compare them with their corresponding native conformations. The RMSD values are less than 1Å (Table 1), indicating the protocol’s ability to replicate correct binding modes under the structural variability.

**TABLE 1:** Cross-docking results of 3 BACE1 structures. RMSD values less than 1Å are considered excellent, within the range of 1-2Å acceptable, while above 2Å are depicted as unreliable results.

Ligand (protein)	2VA7	2VA6	2VA5
L1 (2VA7)	—	0.17	0.26
L2 (2VA6)	0.17	—	0.27
L3 (2VA5)	0.17	0.26	—

### 3.4 Docking and binding pose analysis

**FIGURE 5:**
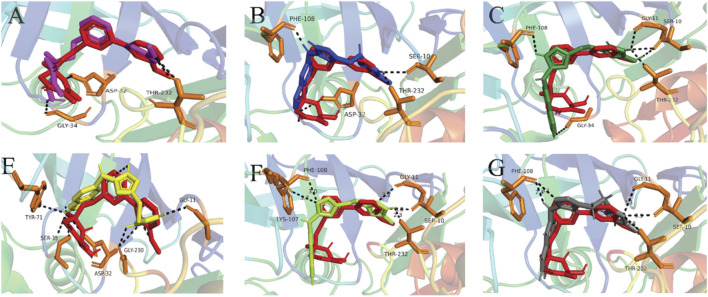
The aligned view with C27 (red color) and binding interactions of labelled key residues of the BACE1 binding pocket with newly designed coumarin compounds such as **(A)** CUM-0199 in deep purple color, **(B)** CUM-0158 in density blue color, **(C)** CUM-0196 in forest green color, **(E)** CUM-0351 in TV yellow color, **(F)** CUM-0421 in lime color, and **(G)** CUM-0350 in grey40 color. Protein secondary structure elements are shown in the background with 50% transparency. Black dashed lines denote the hydrogen bonds, while interacting residues are labelled and displayed as sticks (orange).

After validation of the PyRx docking protocol, 92 hits were subjected to structure-based molecular docking with the BACE1 receptor to acquire a comprehensive understanding of interactions and energies. All docked hits showed favourable binding affinity values between −9.2 and −6.7 kcal/mol (Supplementary Table S4). 34 hits follow the cutoff criteria of −8.0 kcal/mol. The next cutoff was employed based on RMSD (<1.5 Å) along with hydrogen bond distance (<3.5 Å) to pick the best poses and to prioritize binding conformations. Resultantly, the best six hits were selected. These drug-like molecules underwent additional scrutiny through superimposition on the reference ligand (C27), followed by interaction analysis. The results indicate that these chemicals lie within the binding site and depict a similar binding space as observed with C27 (Figure 5).

Various studies reported that Asp32 and Asp228 residues are crucial ones in BACE1’s catalytic mechanism (Edwards et al., 2007). Gly34 was implicated in the glycine loop of BACE1, a pivotal residue involved in substrate recognition and binding. The active site is slightly covered by the flexible flap region of beta-secretase 1. Tyr71 and Phe108, two residues in the flap region, have been linked to substrate binding and catalytic activity (Ghosh and Osswald, 2014).

The binding scores of the best selected compounds, i.e., CUM-0199, CUM-0158, CUM-0196, CUM-0351, CUM-0421, CUM-0350 were observed as −9.2, −8.9, −8.7, −8.7, −8.7, and −8.6 kcal/mol, respectively. On the other hand, the reference compound displays a binding affinity of −8.1 kcal/mol. CUM-0199 displayed the highest binding energy of −9.2 kcal/mol when compared with others and made interactions similar to those of the reference compound. It was observed that polar functional groups of CUM-0199, improved the docking scores by forming stable hydrogen bonds with key catalytic residues, particularly Asp32 and Asp228. Additionally, it made H-bond with polar uncharged residue Thr232. In contrast, hydrophobic substitutions contributed to improved ligand stability through van der Waals interactions with Ser35, Ile118, Ile110, Gly11, Gly13, Gly230, and Trp76 residues. Moreover, it shows nonpolar contacts with the aliphatic Gly34 residue (Figure 5A). On the other hand, the 1,2,3-triazole linker enabled proper interactions, with balanced polar and hydrophobic features, improving docking performance. CUM-0158 makes hydrogen bond interactions with aromatic Phe108, polar uncharged Thr232, Ser10, and negatively charged Asp32 and Asp228 within 3.3Å distance (Figure 5B). CUM-0196 illustrates interactions that lie within 3Å with polar uncharged Ser10, nonpolar aliphatic Gly11, and Phe108 with −8.7 kcal/mol binding affinity (Figure 5C). In Figure 5E, CUM-0351 shows binding pose interactions with Gly11 (2.29 Å), Gly230 (2.3 Å), Tyr71 (2.3 Å), Ser35 (3.2 Å), and Asp32 (3.3 Å). As shown in Figures 5F,G, CUM-0421 and CUM-0350 display H-bonds with Phe108, Gly11, Ser10, and polar uncharged Thr232 within distance cutoff criteria of <3.5 Å. Based on H-bonds, hydrophobic, van der Waals, Pi-Alkyl, Pi-Cation, and anion interactions, the proposed molecules mainly show interactions with conserved active site residues, i.e., Asp32, Gly34, Thr232, Phe108, Tyr71, Ser10, Ser35, Lys107, and Asp228. Table 2 depicts interactions among the active site residues of the receptor and the best 10 hits, along with their distances.

**TABLE 2:** Docking scores with RMSD and some other calculated features of hit compounds.

I Ds	Binding affinities (kcal/mol)	RMSD (Å)	H-bond interacting residues (Å)	van der Waal’s interacting residues (Å)	Pi-Alkyl/Pi-Cation/Anion interacting residues (Å)
CUM-0199	−9.2	0.3	Thr232 (2.2), Asp32 (2.2), Gly34 (2.1), Asp228 (3.3)	Ser35, Ile118, Ile110, Gly11, Gly13, Gly230, Trp76	Tyr71 (3.5), Tyr198 (5), Phe108 (5.2), Trp115 (4.9), Leu30 (5.0)
CUM-0158	−8.9	0.4	Asp32 (2.14), Asp228 (3.3), Gly34 (2.5), Phe108 (1.93)	Trp115, Thr231, Ile126, Arg128, Ser35, Ile118, Leu30, Ile110	Gln12 (3.6), Ile110 (3.7), Ile118 (5), Tyr71 (5), Tyr198 (3.7)
CUM-0196	−8.7	0.5	Phe108 (2.6), Gly11 (2.5), Ser10 (2.12, 2.47)	Ile110, Gly230, Thr231, Gly34, Thr232, Asp32, Gly74	Tyr71 (3.7.4.0), Gln12 (3.6), Phe108 (4.0)
CUM-0351	−8.7	0.5	Gly11 (2.29), Gly230 (2.3), Tyr71 (2.3), Ser35 (3.2), Asp32 (3.3)	Phe108, Ile110, Lys107, Ser10, Gln12, Gly13, Leu30, Thr231, Asp32, Ser35	Ile118 (4.5)
CUM-0421	−8.7	0.5	Gly11 (3.2), Thr232 (2.3), Ser10 (2.01), Lys107 (2.7), Phe108 (3.0)	Gly13, Ala335, Thr231, Ile110, Gly230, Gly34, Ser35, Trp115	Try71 (3.7, 3.9), Gln12 (3.6)
CUM-0350	−8.6	0.6	Lys107 (), Phe108 (2.42, 2.47), Thr232 (2.09), Ser10 (2.5), Gly11 (2.7)	Trp115, Gly13, Lys107, Gly230, Thr231, Asp32, Leu30	Ile110 (3.8), Gln12 (3.7), Ile118 (5.0), Tyr71 (3.9)
CUM-1228	−8.6	0.5	Thr232 (2.7), Ser10 (2.2, 2.5), Gly11 (2.2), Phe108 (2.2, 2.7)	Asp32, Trp115, Lys107, Ser35, Gly230, Thr231, Gln12, Gly13, Ala335	Ile110 (3.7), Ile118 (5.0), Phe108 (4.4)
CUM-0234	−8.5	0.5	Gly11 (2.2), Ser10 (2.2, 2.4), Thr232 (2.6), Gly230 (2.9), Phe108 (2.4, 2.75)	Trp115, Gly13, Gly13, Thr232, Lys107, Asp32, Ala335	Phe108 (4.5), Ile110 (3.9), Tyr71 (3.9, 5.3)
CUM-0736	−8.5	0.5	Asp228	Gly13, Phe108, Asp32, Ser35, Ile110, Thr232, Gly11, Gly13, Ser10	Tyr71 (3.4), Ile118 (4.8), Leu30 (5.0), Gln12 (4)
CUM-1333	−8.5	0.3	Phe108 (2.3, 2.5), Gly11 (2.3), Ser10 (2.4), Thr232 (2.8), Tyr71 (3)	Lys107, Trp115, Leu30, Gln12, Gly13, Gly230, Ala335, Thr231, Asp32, Gly34	Ile110 (3.8), Ile118 (5), Phe108 (4.4)
C27 (Reference ligand)	−8.1	0.2	Gly230 (2.19), Ser10 (3.5), Asp228 (2.7), Asp32 (2.02)	Gly13, Thr232, Ser35, Tyr71, Gly11, Ile118, Thr231, Ile110, Ser229, Ala335	Gln12 (3.9), Leu30 (5), Trp115 (4.9), Phe108 (5)

### 3.5 LE, LLE, and LELP prioritize hit selection

**FIGURE 6:**
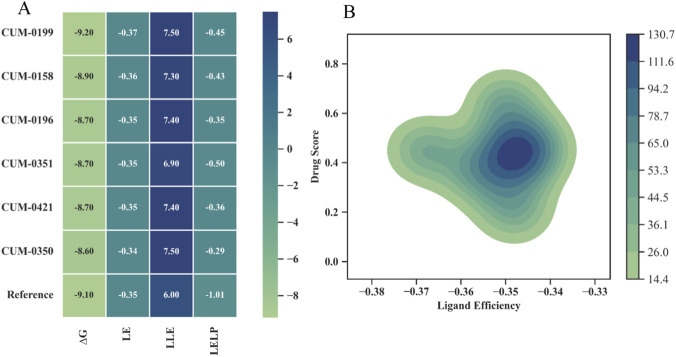
**(A)** Showing comparison of Binding affinity, Ligand efficiency (LE), Lipophilic Ligand Efficiency (LLE), and Ligand Efficiency Lipophilic Penalty (LELP) of the best six hits with the reference ligand C27 **(B)** Representing the correlation between Drug Score and Ligand efficiency of the best coumarin-1,2,3-triazole hybrids and reference compound.

In rational drug design and optimization, ligand efficiency (LE) plays a crucial role in prioritizing hits and identifying potential drug candidates. Commonly, a higher magnitude (more negative) is considered worthy. LE of reference compound was −0.35 kcal/mol. Comparatively, the potency of CUM-0199 relative to its size was −0.37, while for other hits it lies within an acceptable range of −0.34 to −0.36 kcal/mol (Figure 6A). Lipophilic ligand efficiency (LLE) and Ligand Efficiency Lipophilic Penalty (LELP) prioritize lipophilic molecules with favorable drug-like properties, resulting in increased selectivity and overall success in the drug development pipeline. LLE and LELP values for the reference compound were six and -1.01 kcal/mol, respectively. On the other hand, selected compounds contain higher LLE and lower LELP, which are considered beneficial. CUM-0199 contains higher LLE (7.50) and −0.45 LELP, indicating that it achieves binding potency with minimal lipophilicity, which reduces off-target effects and toxicity, better selectivity, and BBB penetration (Hopkins et al., 2014). These matrices are used as preliminary indicators in early-stage drug discovery and further require experimental validation. Figure 6B displays a positive correlation between ligand efficiency and drug score, indicating that high values of LE tend to have better drug scores.

### 3.6 ADME and toxicity profile of potential compounds

**FIGURE 7:**
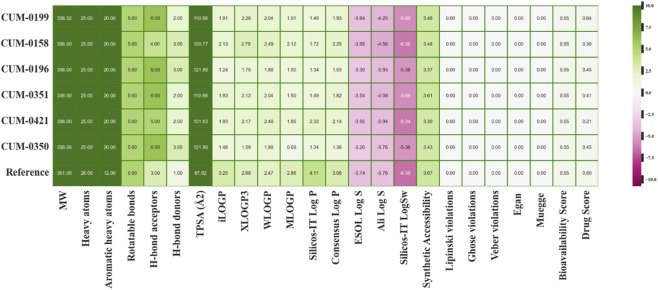
Physicochemical properties of the best six hits along reference compound C27. These molecules have suitable physicochemical properties: molecular weight (MW) between 300 and 400 Da, hydrogen bond acceptor (HBA) ≤5, hydrogen bond donor (HBD)≤10, LOGP ≤5, topological polar surface area (TPSA) < 140, number of rotatable bonds ≤15, heavy atoms (HA), and aromatic heavy atoms (AHA).

To investigate potential therapeutic candidates, the approach of drug-likeness evaluates hits based on their physicochemical properties, structural features, ADMET analysis, and specific rules, i.e., Lipinski, Egan, Veber, Muegge, and Ghose. As seen in Figure 7, physicochemical properties of all the selected hits lie within the acceptable range as stated in the rule of five (RO5), and no compound violates these rules.

CNS (central nervous system) drugs have stringent criterion than peripheral targets. Therefore, rigorously inspect all the checkpoints as listed in Table 3. Absorption level of hits was predicted via Caco-2 (log cm/s), MDCK, skin, and GI (gastrointestinal) absorption parameters, along with 20% and 30% human oral bioavailability. Hits considered Caco-2 Permeable with predicted value > -5.15 log cm/s. CUM-0199, CUM-0351, CUM-0421 compounds showed excellent absorption levels. All hits demonstrate moderate to good MDCK Permeability between the range of 4e-06 to 6.7e-06 cm/s. The drug should have adequate penetration through the skin to attain therapeutic concentration while avoiding excessive systemic absorption. According to predicted data, all hits showed skin permeation. High GI absorption is frequently required for oral medications, and all hits fulfill this criterion. Human oral bioavailability is an essential pharmacokinetic parameter that should be ≥ F20% and ≥ F30%, within the range of 0–one (excellent to poor). CUM-0199 and CUM-0421 compounds depict higher F20% and F30% values, indicating better oral bioavailability, while CUM-0158 and CUM-0196 compounds show moderate bioavailability. Contrastingly, CUM-0350 displays lower F20% and F30% values. The CNS drugs should be BBB (blood-brain barrier) permeant to reach their targeted receptor, and the value should lie within 0–one (excellent to poor) range. All hits displayed BBB permeability except CUM-0158 and CUM-0421. Other distribution parameters, such as PPB (plasma protein binding) and volume distribution (VD), describe *In vivo* drug uptake and distribution. Hits with higher PPB and VD above the range of 0.04–20L/kg exhibit a lower therapeutic index. CUM-0199 shows a lower PPB value than others, along with an excellent 0.765L/kg VD. Phase I human cytochrome P450 (CYPs) enzymes are drug metabolizing enzymes (oxidative reactions) that bio-transform and detoxify >80% xenobiotics and values lie within the range of 0–1. All hits are prime CYP1A2 inhibitors within the range of 0.7–1.0. Moreover, 50% hits, i.e., CUM-0199, CUM-0351, and CUM-0421 were moderate to effective CYP2C19, CYP2C9, CYP2D6, CYP3A4 inhibitors, and could be used as chemotherapeutic agents. Another significant pharmacokinetic metric is drug clearance (CL), which is crucial for reducing toxicity and improving the therapeutic efficacy of hits. Predicted CL value exceeding 5 mL/min/kg signifies excellence, while CL < 5 mL/min/kg indicates poor clearance. Reported data for all compounds denoted moderate CL values < 15 mL/min/kg, which clearly showed that compounds eliminated from excessive clearance criteria and did not demonstrate toxicity. Half-life of all hits along the reference system ranges from 0 to one (excellent to poor), indicating reduced risk of accumulation and sustained therapeutic effect.

**TABLE 3:** ADMET analysis of the best selected compounds along with some toxicophoric rules’ alert. The prediction values transformed into six symbols: 0-0.1(---), 0.1-0.3(--), 0.3-0.5(−), 0.5-0.7(+), 0.7-0.9(++), and 0.9-1.0(+++). MDCK (Madin−Darby Canine Kidney cells), Caco-2 (human colon adenocarcinoma cell lines), BBB (blood-brain barrier), PPB (plasma protein binding), F20%, F30% (oral bioavailability).

Pharmacokinetic properties	​	CUM-0199	CUM-0158	CUM-0196	CUM-0351	CUM-0421	CUM-0350
Absorption	Caco-2 permeability	−5.121	−5.230	−5.311	−5.097	−5.080	−5.351
​	MDCK permeability	5.4e-06	4e-06	4.7e-06	6.7e-06	5.3e-06	5.1e-06
​	Skin permeation (cm/s)	−6.74	−6.37	−7.12	−6.86	−6.86	−7.23
​	GI absorption	High	High	High	High	High	High
​	F_20%_	0.1	0.2	0.5	0.7	0.1	0.7
​	F_30%_	0.1	0.5	0.8	0.9	0.2	1.0
Distribution	BBB penetration	0.3	0.5	0.3	0.3	0.7	0.3
​	PPB	94.168%	95.540%	95.233%	95.229%	95.166%	95.055%
​	Volume distribution	0.765	0.894	1.040	0.656	1.425	0.816
Metabolism	CYP1A2 inhibitor	+++	+++	++	+++	+++	++
​	CYP2C19 inhibitor	+	+	--	+	++	-
​	CYP2C9 inhibitor	--	---	-	---	+	--
​	CYP2D6 inhibitor	+	--	-	+	+	-
​	CYP3A4 inhibitor	-	-	--	--	+	--
Excretion	Total clearance (mL/min/kg) T_1/2_	8.975 0.192	8.074 0.258	7.642 0.413	10.000 0.277	6.895 0.282	8.529 0.779
Toxicity	hERG blockers	---	--	--	-	--	-
​	Human hepatotoxicity	--	+++	-	+++	+	-
​	Drug-induced liver injury	++	+++	+++	+++	+++	+++
​	AMES toxicity	--	-	--	--	--	---
​	Rat oral acute toxicity	--	++	--	++	-	++
​	Eye irritation	--	--	---	---	---	---
​	Eye corrosion	---	---	---	---	---	---
​	Respiratory toxicity	+	+++	+	++	-	+
​	Skin sensitization	--	+	---	+	--	--
​	Carcinogenicity	+++	+++	+++	+++	+++	++
​	Mutagenic	No	No	No	No	No	No
​	Tumorigenic	No	No	No	No	Yes	No
​	Reproductive effect	No	No	No	No	Yes	No
​	Irritant	No	No	No	No	No	No
Toxicophoric rules	Acute toxicity rule	0 alert	0 alert	0 alert	0 alert	0 alert	0 alert
​	Genotoxic carcinogenicity rule	0 alert	2 alerts	5 alerts	1 alert	1 alert	1 alert
​	Nongenotoxic carcinogenicity rule	0 alert	0 alert	1 alert	0 alert	1 alert	1 alert
​	Aquatic toxicity rule	0 alert	0 alert	3 alerts	0 alert	0 alert	1 alert
​	Non-biodegradable rule	0 alert	0 alert	0 alert	0 alert	1 alert	1 alert
​	SureChEMBL rule	0 alert	1 alert	0 alert	0 alert	0 alert	0 alert

The compounds frequently fail and lead to costly termination of the drug development process due to their toxicity profile. Thus, it is preferable to foresee it at the early stages. None of the hits acts as hERG blockers, indicating that they do not inhibit the normal function of the potassium channel. Three hits demonstrated excellent hepatotoxicity (H-HT) while two exhibited moderate toxicity, and only CUM-0199 was nontoxic. One of the primary safety concerns of hit withdrawal from the market is DILI (drug-induced liver injury), and the probability of DILI was very low for all hits. None of the compounds demonstrated Ames toxicity, eye irritation, eye corrosion, or mutagenicity, while 50% depicted oral rat acute toxicity. In addition to this, CUM-0421 exhibited tumorigenic and reproductive effects. Further hits filtered based on toxicophoric rules, i.e., acute toxicity rule, genotoxic carcinogenicity rule, nongenotoxic carcinogenicity rule, nonbiodegradable rule, aquatic toxicity rule, and Sure-ChEMBL rule (Alabbas, 2023). Solely CUM-0199 was identified as the potential hit among the top compounds as it passes all these rules.

### 3.7 Bioactivity profile analysis of potent hits

Bioactivity scores better comprehend compound’s poly-pharmacological potential and identify potential hits along with a favorable safety profile. The compounds with poor bioavailability may be unable to access their target site in sufficient concentrations to achieve a therapeutic effect and may require modifications to improve their bioavailability and activity. A compound is deemed active if its bioactivity score exceeds 0.0, slightly active if it falls within −5.0 and 0.0, and inert if it falls below −5.0. Table 4 displayed ​that all hits had bioactivity scores ranging from −5.0 to 0.45, clearly indicating their potential as drugs.

**TABLE 4:** Bioactivity score of the top six compounds with different human receptors.

Parameters of bioactivity score						
Compounds	GPCR ligand	Ion channel modulator	Kinase inhibitor	Nuclear receptor ligand	Protease inhibitor	Enzyme inhibitor
CUM-0199	−0.35	−0.54	−0.03	−0.70	−0.48	−0.10
CUM-0158	−0.33	−0.32	0.06	−0.50	0.45	−0.10
CUM-0196	−0.20	−0.63	0.08	−0.80	−0.55	−0.07
CUM-0351	−0.35	−0.54	−0.03	−0.70	−0.48	−0.10
CUM-0421	−0.20	−0.56	0.18	−0.83	−0.38	−0.12
CUM-0350	−0.06	−0.21	0.18	−0.70	−0.33	0.04

### 3.8 Structural stability of hits via density functional theory

**FIGURE 8:**
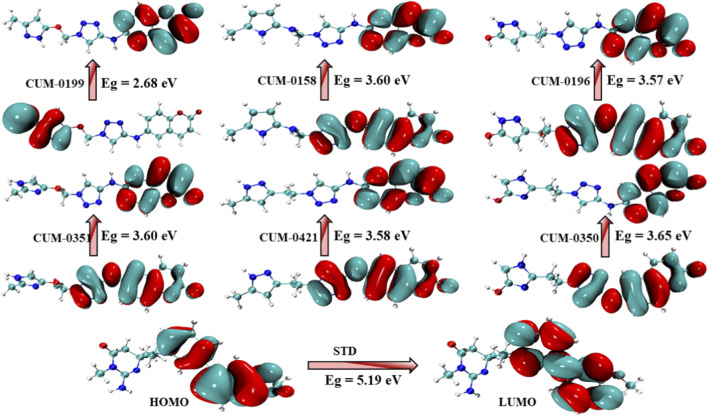
Electron density region distributions within the HOMO-LUMO range were analyzed via the B3LYP/6-31G**.

In comparison to other investigated compounds, CUM-199 exhibits a significantly lower energy gap (E.g.,). The interactions between ligands and proteins in a complex primarily depend on FMOs. Favourable FMO distribution facilitates H-bonding and electrostatic interactions with BACE1 active site residues such as Asp228 and Asp32. The decreasing order of energy gap values was STD > CUM-0350 > CUM-0351 > 0158 > CUM-0421 > CUM-0196 > CUM-0199. Specifically, the energy gap values for CUM-0199, CUM-0158, CUM-0196, CUM-0351, CUM-0421, CUM-0350, and STD were 2.68 eV, 3.60 eV, 3.57 eV, 3.60 eV, 3.58 eV, 3.65 eV, and 5.19 eV, respectively. CUM-0199 exhibited the greatest reactivity in terms of charge excitation, excitation values, energy gap, and the smallest LUMO level, owing to the inverse correlation between the amount of energy difference and ligand reactivity. Higher reactivity may enhance the ligand’s ability to form satisfactory interactions with the BACE1 active site, thus establishing stable protein-ligand complex formation. The negative values of the chemical potential (µ), as presented in Table 5, signify that all compounds are stable. The chemical potential reflects the tendency of the acceptor to acquire a negative charge, which indicates the stability of the compounds and their capacity to form enduring complexes with the receptor (Kumar et al., 2023). In comparison to the other compounds, CUM-0199 stands out with a hardness (ղ) value of 1.34, making it well-suited for further analysis. This observation aligns with its docking trend and potential connectivity. The electron density is depicted graphically in Figure 8 using the HOMO-LUMO orbitals. Red and cyan were used to represent the HOMO and LUMO states, respectively. These colors aid in locating areas rich in electrons. It becomes clear that CUM-0199 has the best inhibitory qualities after an extended examination of HOMO, LUMO, and complete reactivity indicators. This is because it has a small energy gap, which improves local and global attributes.

**TABLE 5:** Calculated energy of HOMO and LUMO, energy gap (E.g.,), Hardness (*η*), Softness (S), electronegativity (X), chemical potential (µ), and electrophilicity index (ɷ) of all investigated compounds.

Compounds	HOMO	LUMO	E.g.,	η	μ	S	χ	ω
CUM-0199	−4.625	−1.938	2.68	1.34	3.28	0.74	−3.28	4.01
CUM-0158	−5.478	−1.870	3.60	1.80	3.80	0.55	−3.80	4.00
CUM-0196	−5.403	−1.825	3.57	1.78	3.61	0.56	−3.61	3.65
CUM-0351	−5.453	−1.844	3.60	1.80	3.64	0.55	−3.64	3.67
CUM-0421	−5.407	−1.824	3.58	1.79	3.61	0.55	−3.61	3.57
CUM-0350	−5.466	−1.808	3.65	1.82	3.63	0.54	−3.63	3.61
STD	−6.011	−0.812	5.19	2.59	3.41	0.38	−3.41	2.23

#### 3.8.1 Molecular electrostatic potential (MEP)

**FIGURE 9:**
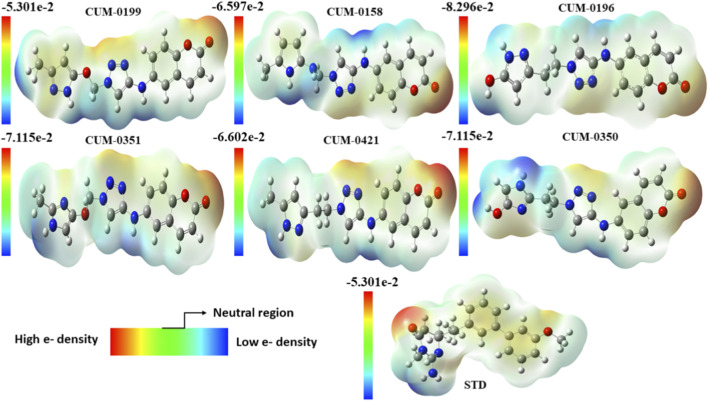
MEP maps illustrate the locations where investigated compounds are susceptible to electrophilic and nucleophilic attacks.

Understanding the distribution of electrons in drug discovery systems and studying how molecules react to electrophilic and nucleophilic assaults are both aided by MEP maps. As shown in Figure 9, the MEP surface map provides a visual representation of the regional distribution of molecular charges. These representations draw attention to areas of changing charge density inside molecules, shedding information on their interactions with biological entities through hydrogen bonding and exposing changes in electron density. It is now feasible to predict molecular interactions by evaluating data on charge distributions. In addition, the amount of positive and negative charges a molecule carries may be deduced from the charge density areas of the MEP map (Abbas et al., 2023b). This property is widely used in a variety of contexts, including the prediction of electrophilic attack sites, the measurement of relative reactivity, the analysis of biological recognition, and the investigation of interactions enabled by hydrogen bonding or electrostatic interactions. Polar and negatively charged regions are indicated in red for molecular electric density, whereas non-polar and positively charged regions are shown in blue. On the many faces of the (MEP) maps, different colors represent different electrostatic potentials (Kiran et al., 2021). Our developed compound, CUM-0158, demonstrates a robust capacity for charge transfer, as assessed through the global reactivity parameter known as chemical potential (3.80 eV). The charge transfer properties of the molecule are heavily impacted by its chemical potential value. It should be noted that the chemical’s potential value is proportional to the amount of charge transfer. Compound CUM-0158 shows a high electronegativity (X = 3.80), low hardness, high softness, ideal nucleophilic attacking index, and the most favorable level of energy (LUMO level −1.870 eV), hence indicating that it is electrophilic, according to the investigation. Another important reactivity measure, hardness, is calculated by comparing the energy gaps among the HOMO and LUMO orbitals. A large energy gap represents a stiff molecule, whereas a small one is indicative of a flexible molecule linked to very reactive chemical compounds.

The MEP maps exhibit a color scheme where blue signifies advantageous positions for nucleophilic attacks, red denotes areas unfavorable for electrophilic attacks, and green indicates sites with zero potential or neutral regions between the highly negative and positive regions within the studied compounds.

#### 3.8.2 Density of state analysis (DOS)

**FIGURE 10:**
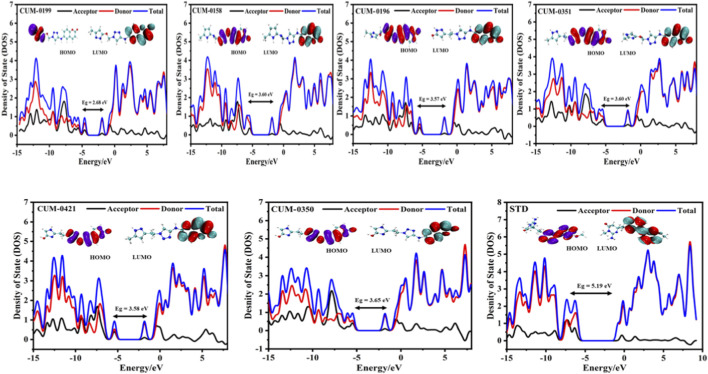
The density of each ligand in the DOS spectra, plotted at Isovalue 0.0004, illustrates the individual contribution of each fragment.

Figure 10 depicts the results of a density of state analysis, which measures the total number of states in a system over a specific energy range. This evaluation is important for analyzing the interactions between fragments and their impact on the energy levels of molecular orbitals in the complex. DOS was evaluated using the B3LYP/6-31G** theoretical framework to understand the influence of each segment on the charge density distribution order. The FMO and DOS are Frontier molecular orbitals. The density of electron distribution in these orbitals alters when acceptor components with varying electron-withdrawing capacities are used, according to further investigations on the ratios of different components in HOMO and LUMO (Lee, 2020). Occupying the charge distribution surrounding the donor and acceptor groups are bonding and antibonding orbitals, as seen in the DOS diagram. Negative energy values represent bonding orbitals, and positive energy values represent antibonding orbitals. A score of 0 means there is no contact between the bonding orbitals. DOS calculations confirmed the FMO observations. Each molecule exhibits a specific arrangement of the acceptor groups HOMO and LUMO, which results in a change in the energy difference between these two orbitals. The extent of this interaction can be measured by examining the DOS spectrum.

### 3.9 Conformational stabilities of potent hit complexes via MD simulation

**FIGURE 11:**
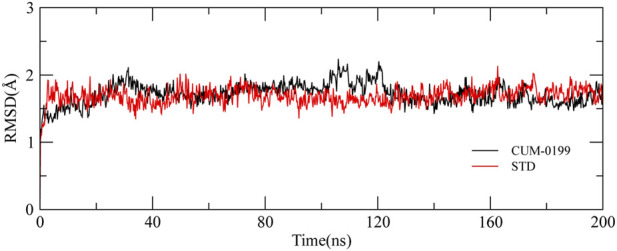
RMSD of Ca atoms of CUM-0199-BACE1 system displayed in black color, while the comparative STD system (BACE1 in contact with 2va7) is displayed in red color and shows minimal variations over time. The Y-axis depicts RMSD values, while the X-axis delineates time in nanoseconds.

Through investigation of 200 ns MD simulation, assess the overall stability, flexibility, inter-molecular interactions, and accuracy of both complex conformations (CUM-0199 and STD systems). Figure 11 illustrates the RMSD for the best hit and the standard (STD) system’s carbon alpha atoms. Generally, the optimal range for stable complexes, in terms of RMSD, is considered to be between 2 and 3 Å. It is noteworthy that both complexes exhibit stable P-RMSD (Protein Root Mean Square Deviation) values, lie within 1.8 ± 0.05 Å, and remain steady with little variation over the simulation time. Protein RMSD of CUM-0199 system stabilizes till 100ns, followed by a little upsurge until 120ns, and subsequently re-establishes stability until 200ns. P-RMSD of the standard (STD) system remains steady over the period of simulation. Ligand RMSD varied within the range of 2 Å, indicating that the ligand retained its binding mode in the active site of BACE1 and did not undergo large conformational changes.

**FIGURE 12:**
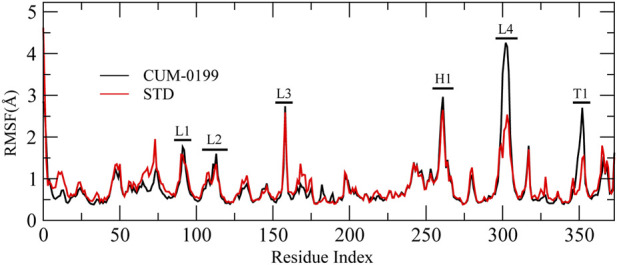
Residue-wise RMSF of targeted receptor (PDB ID: 2VA7) in contact with CUM-0199 (black color) and reference molecule (red color). L demonstrates loops, H depicts helices, and T represents turns.

In order to check the mobility of both complex systems, the Root Mean Square Fluctuation (RMSF) of individual residues was calculated across the simulation time. Figure 12 indicates regions that experience significant fluctuations during simulation. An elevated RMSF value indicates flexible regions, i.e., loops, while a lower RMSF value demonstrates rigid segments such as alpha helices and beta sheets. Specific elevated regions, such as L1 to L4, demonstrate loops, H1 depicts helices, and T1 represents turns. The N-terminal region of the receptor in both complex systems exhibits heightened flexibility due to the existence of loops and exposure to the solvent environment. Apart from that, both CUM-0199 and STD systems maintain a lower RMSF value throughout the simulation time. Active site residues, i.e., Thr232, Asp32, and Gly34, depict minimal mobility, signifying that conformational alterations within these regions are minimal. These observations imply secure and firm binding of CUM-0199 compound within the active site of the targeted receptor. To get an understanding of mobility in the studied systems, P-RMSF was compared with the experimental B-factor value (Kuzmanic and Zagrovic, 2010). An average B factor value was reported as 33.39 Å^2^ (∼5.77 Å), indicating average thermal motion of atoms in the crystal structure. On the other hand, both simulated complexes display almost the same trend in RMSF values as they lie within an optimal range of ∼2 to 2.9 Å, except for one loop (L4) region. Residues in the range of 295–310 exhibited greater fluctuations compared to other residues. Overall, both complexes depict acceptable RMSF values, indicating reduced mobility and competent system stability.

#### 3.9.1 Exploration of secondary structure elements and stability analysis

**FIGURE 13:**
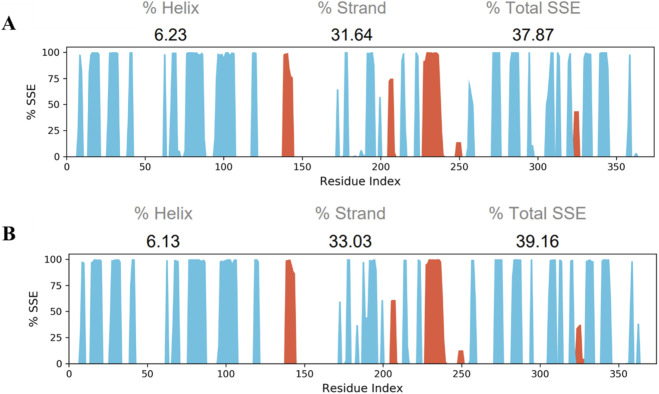
SSE distribution by residue index throughout the protein structure in both **(A)** CUM-0199 and **(B)** STD system, along secondary structure component percentages. Cyan represents beta-strands, while orange color depicts helices.

Analysis of secondary structure elements (SSEs) provides crucial insights about structural characteristics and stability profile of complexes. The distribution of SSEs in both complexes was explored to track conformational changes and the influence of the attached ligand on the secondary structure formation (α-helices and β-sheets), which is considered crucial for their functionality. The pattern of alpha helices formation remains almost stable with only minor variations, as seen in Figure 13, while the pattern of beta sheets fluctuates ∼2% during the entire simulation period in both CUM-0199 and STD systems. CUM-0199 system is made up of 6.23% alpha helices, 31.64% beta strands, and a total of 37.87% SSEs. On the other hand, the STD system constitutes 39.16% of total SSEs with 6.23% alpha-helices and 39.16% beta strands. Secondary structure analysis revealed that the percentage of β-sheet decreases in CUM-0199 as compared to the STD system. We noticed that the attached ligand somewhat impacted the formation of secondary structures and reduced the content of stable moieties like beta strands, but this little change in SSEs does not leave a huge impact on the 3D structure and function of the targeted receptor.

#### 3.9.2 Comprehensive dynamic interactions

**FIGURE 14:**
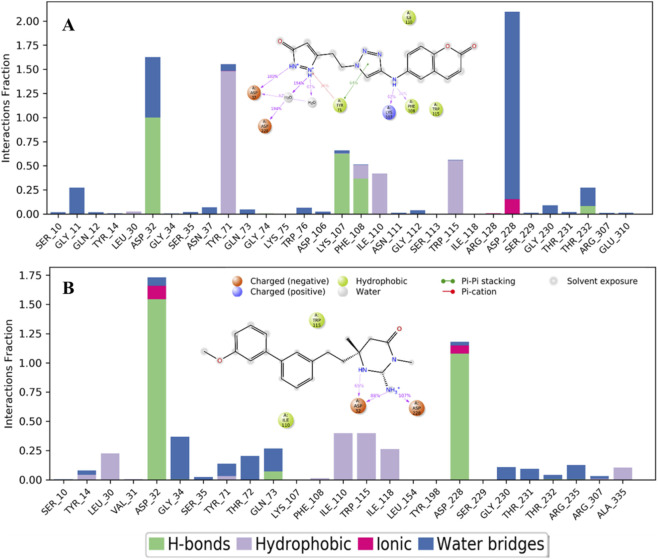
**(A)** Summary of all contacts between BACE1 and CUM-0199, while **(B)** shows interaction information of reference molecule C27 and targeted receptor during MD simulation. Additionally, it represents protein-ligand interactions for both systems, which occur over the simulation period.

Figure 14 depicts a summary of several types of interactions that CUM-0199 and STD (C27) compounds had with the receptor over the simulation time. CUM-0199 and STD made H-bonds, hydrophobic, water bridges, and ionic interactions with BACE1’s active site residues. H-bonds hold paramount significance among other types of interactions. In the case of the current simulation study, hydrogen bonds and water bridges are the major types of interactions, while hydrophobic and ionic interactions contribute around 64% and 36% of the simulation time, respectively. Negatively charged Asp32 and Asp228 are important residues to their activity, and they make multiple interactions such as H-bonds, ionic, and water bridges, over 100% and 194% of simulation time. Based on the protein-ligand contact summary of both systems, an average H-bond formation capacity was higher in the Asp32 residue compared to others. Positively charged Lys107, aromatic Phe108, and Tyr71 depict H-bonds, water bridges, and hydrophobic interactions for more than 62% of the simulation period. Gly34, Ser35, Thr232 made water bridges while Ile110, Trp115, Ile118 made hydrophobic interactions with CUM-0199 and STD compound C27. Strength and number of interactions of the mentioned residues within the active site of BACE1 depict the stability of both complexes.

**FIGURE 15:**
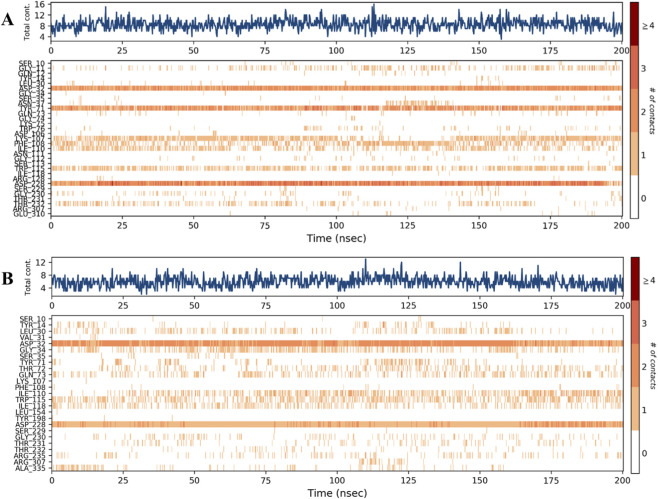
A Timeline representation of protein-ligand contacts with **(A)** CUM-0199 and **(B)** reference molecule in each trajectory frame. Residues make more than one specific contact with the ligand represented by a darker shade of orange, according to the scale to the right of the plot.

Chronological representation of the contacts established by CUM-0199 and STD compounds with residues of the target protein, illustrated in Figure 15. The cumulative count of receptor-ligand interactions was displayed in the top panel, while the bottom panel lists the most significant and persistent forms of interactions formed by BENZI-0660 and STD. Robust interactions with CUM-0199 and STD are indicated by the abundance of deep orange bands in the bottom panel, which correspond to certain residues, i.e., Asp32, Tyr71, and Asp228.

#### 3.9.3 Ligand torsion profile of CUM-0199 and STD system

**FIGURE 16:**
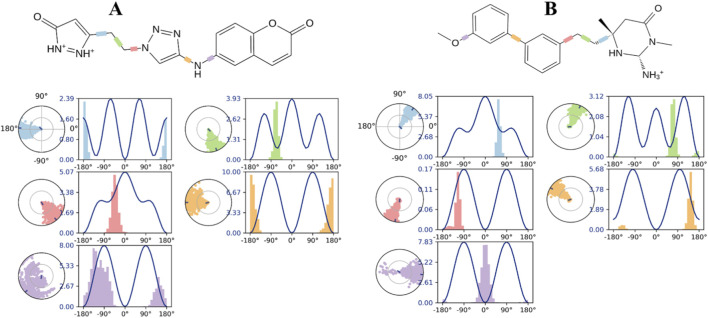
**(A)** Ligand torsion plot of CUM-0199 represents conformational evolution of the ligand’s rotatable bonds throughout the simulation **(B)** Ligand torsion profile of reference molecule. The top panel represents the 2 days scheme of CUM-0199 and STD. All rotatable bonds are shown with different colors. Bottom panel shows dial plot and bar plots, and the values of potential are on the left Y-axis of the chart, represented in kcal/mol.

Ligand torsion profile of CUM-0199 and STD ligands illustrated in Figure 16 over 200 ns MD simulation, which gives insights about the conformational stretch that ligands undergo to retain receptor-bound conformation (Kalasariya et al., 2022). The top panel for both studied ligands displays five rotatable bonds (RBs) in distinct colors, whereas the bottom panel shows the dial and the bar plots in the same color. Average probability density for CUM-0199 lies near 180°, 70°, −120°, −80°, and −180° with 2.39, 5.07, 8.00, 3.93, and 10.00 kcal/mol torsion potential, respectively. On the other hand, STD depicts average probability density around 80°, −100°, 0°, 80°, 150° with torsion potential of 8.05, 0.17, 7.83, 3.12, 5.68 kcal/mol, respectively. Table 6 demonstrates both studied ligands’ properties. RMSD for CUM-0199 lies between 0.6–1.8 Å, while for STD ranged between 0.8–1.2 Å. The radius of gyration (rGyr) provides insights into the conformational flexibility of ligands. Larger rGyr fluctuations indicate greater conformational changes. rGyr for CUM-0199 and STD lies within the optimum range of 4.00–4.50 Å. Molecular surface area (MolSA) for CUM-0199 exhibits dynamic variations between 304–312 Å^2,^ while for STD it diverges from 344 to 360 Å^2^. Solvent Accessible Surface Area (SASA) for CUM-0199 and STD demonstrates fluctuation spanning from 50–200 Å^2^ and 30–120 Å^2^, respectively. Polar Surface Area (PSA) varies from 240–258 Å^2^ and 108–126 Å^2^ for CUM-0199 and STD, respectively.

**TABLE 6:** Both CUM-0199 and STD compounds’ properties were determined via MD simulation, such as RMSD, Molecular Surface Area (MolSA), Radius of Gyration (rGyr), Polar Surface Area (PSA), and Solvent Accessible Surface Area (SASA) fluctuated near the equilibrated state, confirming the stability of both ligands.

Ligand properties	RMSD (Å)	MolSA (Å^2^)	SASA (Å^2^)	PSA (Å^2^)	rGyr (Å)
CUM-0199	1.2	310	110	247	4.45
C27 (Reference)	1.2	355	70	120	4.25

### 3.10 Binding free energy analysis

**FIGURE 17:**
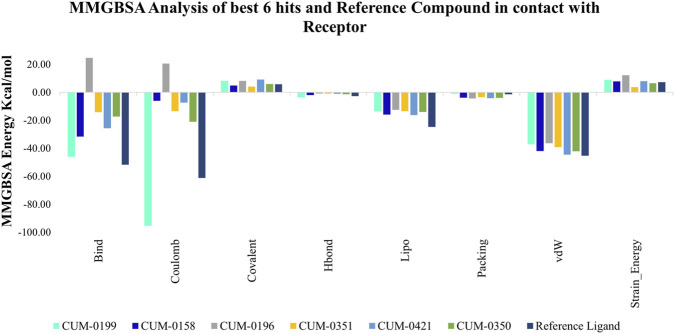
MM-GBSA analysis of the best six hits and reference ligand with BACE1 receptor along numerous energies, i.e., Binding free energy, Coulombic, covalent, hydrogen binding energy, lipophilic, Generalized Born electrostatic solvation energy, and van der Waals binding energy.

The binding free energy of both complex systems was computed via Molecular Mechanics-Generalized Born Surface Area (MM/GBSA) analysis, which helps to assess the strength of protein-ligand interaction, confirm the accuracy of best ligand poses, and binding orientations. and rank ligands based on their binding free energies (ΔGbind). All these constraints are beneficial for the systematic design and development of a drug. MM/GBSA calculates ΔGbind to offer reliable estimates of binding affinity, as shown in Figure 17, which depicts the binding free energy landscape for six hits in comparison to the reference system. According to the energy landscape, CUM-0199 has a more pronounced negative ΔGbind = −46.03 kcal/mol value, comparable to STD’s ΔGbind = −51.76 kcal/mol value, indicating robust ligand-receptor interactions. The variation in ΔGbind values between reported and virtually designed hits could be due to size differences. Van der Waals and Coulombic interactions contribute more to all systems than other types of interactions. CUM-0158, CUM-0196, CUM-0351, CUM-0421 systems display −31.65, 24.74, −14.21, −25.69, −17.30 ΔGbind values, respectively. MM/GBSA results were consistent with molecular docking and MD simulation results, and the ΔGbind value supports CUM-0199 to inhibit the BACE1 receptor.

## 4 Conclusion

In the current study, shape and distance-based virtual screening techniques identified tentative hits featuring a 1,2,3-triazole linking group and a coumarin fragment. Designed coumarin-1,2,3-triazole hits docked well in BACE1’s active site and displayed tremendous pharmacokinetic properties. These compounds indicate substantial improvements in the development of non-peptidic, druglike BACE1 inhibitors with moderate to excellent LE, bioavailability, and bioactivity. DFT studies gave excellent insights into the steric and electrical properties of top-selected compounds, important in their biological activity, and assessed the possibility of hits as therapeutic candidates. MD simulation and binding free energy analysis suggested that CUM-0199 system depicts stability along acceptable conformational changes with −46.03 kcal/mol binding free energy. Asp32 and Asp228 residues play a significant role in the binding of identified hits with the BACE1 receptor. We proposed that such information could be employed to develop a medication that selectively targets BACE1 while not disrupting other biological functions. The results will be useful to researchers and may lead to the development of a novel medicine for the treatment of Alzheimer’s disorders.

## Data Availability

The original contributions presented in the study are included in the article/supplementary material, further inquiries can be directed to the corresponding author.
