# Supplementary material for: Unveiling the structural insights and inhibitory potential of coumarin-1,2,3-triazole hybrids against BACE1: a promising approach for Alzheimer’s disease therapy
Source: Front Chem. 2026 Jul 7;14:1824875. doi: 10.3389/fchem.2026.1824875 (PMC13385185; doi:10.3389/fchem.2026.1824875)
Supplement: Supplementary file 1 [file Supplementaryfile1.docx]

Table S1: Co-crystal ligands along their PDB IDs, formula, biological activity in nM, molecular weight, and physicochemical properties such as hydrogen bond acceptor, hydrogen bond donor, and logp.

| **No.** | **Cocrystal ligand** | **PDB ID** | **Structure** | **Formula** | **IC_50_ / KI nM** | **Molecular weight** | **H.B.A.** | **H.B.D.** | **Log P** |
| --- | --- | --- | --- | --- | --- | --- | --- | --- | --- |
| 1 | CMZ | 2OF0 |  | C_15_H_23_NO_3_ | 1.00e+6 | 265.35 | 4 | 1 | 1.7 |
| 2 | 4FP | 2OHN |  | C_12_H_16_FN | 12 | 193.26 | 2 | 1 | 2.6 |
| 3 | 6IP | 2OHP |  | C_15_H_15_N_3_ | 9.40e+4 | 273.30 | 2 | 2 | 2.9 |
| 4 | 9IP | 2OHS |  | C_18_H_18_N_4_O | 4.00e+4 | 306.4 | 5 | 2 | 2.5 |
| 5 | IP6 | 2OHT |  | C_20_H_18_N_4_ | 9100 | 313.4 | 3 | 3 | 3.7 |
| 6 | 7IP | 2OHQ |  | C_20_H_20_N_2_O | 2.50e+4 | 304.4 | 3 | 1 | 4.4 |
| 7 | MMI | 1XS7 |  | C_38_H_62_N_6_O_8_ | 25.1 | 730.9 | 8 | 7 | 4 |
| 8 | 5E7 | 5DQC |  | C_36_H_49_N_5_O_7_S | 45.6 | 695.9 | 9 | 6 | 3 |
| 9 | BSD | 2VKM |  | C_36_H_42_N_4_O_6_S | 1.8 | 658.8 | 8 | 4 | 4 |
| 10 | 0GH | 4GID |  | C_35_H_47_N_5_O_6_S | 99 | 665.8 | 8 | 5 | 3.6 |
| 11 | IP7 | 2OHU |  | C_26_H_23_N_5_O | 4200 | 421.5 | 5 | 3 | 4.2 |
| 12 | 1SQ | 2OHK |  | C_9_H_8_N_2_ | 2.00e+6 | 144.17 | 2 | 1 | 1.9 |
| 13 | 2AQ | 2OHL |  | C_9_H_8_N_2_ | 9.00e+5 | 144.17 | 2 | 1 | 1.9 |
| 14 | C8C | 2VA5 |  | C_14_H_14_N_4_O | 8.60e+4 | 254.29 | 2 | 3 | 1.1 |
| 15 | H24 | 2VA6 |  | C_19_H_21_N_3_O_2_ | 670 | 323.4 | 3 | 1 | 2 |
| 16 | C27 | 2VA7 |  | C21 H25 N3 O2 | 1.5 | 351.4 | 3 | 1 | 2.7 |
| 17 | DBO | 2EWY |  | C33 H32 N2 O3 | 700 | 504.62 | 4 | 3 | 5.8 |
| 18 | 66H | 5HU0 |  | C21 H18 N4 O3 | 595 | 374 | 4 | 2 | 2.2 |
| 19 | WZV | 3ZKI, 2ZKN, 3ZKS |  | C20 H20 F3 N5 O3 | 1200 | 435.40 | 8 | 2 | 1.6 |
| 20 | 6T9 | 3ZLQ |  | C19 H19 F3 N4 O3 | 904 | 408.4 | 8 | 2 | 2.3 |
| 21 | C7O | 6JSZ |  | C21 H23 F2 N5 O4 S2 | 1.9 | 511.6 | 10 | 2 | 1.5 |
| 22 | 66F | 7D5B |  | C17 H17 F2 N5 O3 S | 11 | 409.7 | 7 | 2 | 0.6 |
| 23 | FRP | 2FDP |  | C33 H41 F N4 O3 | 26 | 560 | 5 | 3 | 5.4 |
| 24 | MY9 | 2P8H |  | C33 H40 F N5 O6 S | 71 | 653 | 9 | 4 | 3.2 |
| 25 | QIN | 2OAH |  | C24 H37 N5 O3 S2 | 11 | 507 | 8 | 3 | 3.5 |
| 26 | 8AP | 2OHM |  | C12 H13 N3 | 3.10e+5 | 199.25 | 3 | 2 | 2 |
| 27 | L01 | 1W51 |  | C32 H41 N3 O4 | 500 | 531.7 | 5 | 3 | 4.9 |
| 28 | P6U | 5T1U |  | C16 H18 F5 N3 S | 69 | 379.4 | 8 | 2 | 3.1 |
| 29 | 5MS | 3EXO |  | C18 H18 N4 O2 S | 2.40e+4 | 354.4 | 6 | 2 | 3.1 |
| 30 | 5HA | 2B8L |  | C31 H38 N4 O5 S | 233 | 578.7 | 7 | 4 | 3.6 |
| 31 | 3BN | 2B8V |  | C29 H33 N3 O5 S | 98 | 535.7 | 7 | 3 | 3.8 |
| 32 | I02 | 2IRZ |  | C28 H30 F N5 O4 S | 12 | 551.6 | 9 | 2 | 2.9 |
| 33 | I03 | 2ISO |  | C28 H32 F N3 O6 S | 200 | 557.6 | 9 | 3 | 2.4 |
| 34 | 310 | 2ZDZ |  | C27 H23 Cl N4 O3 | 700 | 486.9 | 3 | 2 | 4.9 |
| 35 | 411 | 2ZE1 |  | C26 H22 Br N5 O2 | 600 | 516.4 | 2 | 3 | 4.6 |
| 36 | 879 | 3L38 |  | C26 H20 Cl N5 O | 100 | 453.9 | 5 | 1 | 4.6 |
| 37 | LIJ | 2HIZ |  | C36 H49 N3 O7 S | 112 | 667 | 8 | 4 | 5.2 |
| 38 | 8IP | 2OHR |  | C17 H16 N4 | 1.00e+5 | 276 | 4 | 2 | 2.5 |
| 39 | LIQ | 2HM1 |  | C33 H42 F2 N4 O5 S | 150 | 644 | 9 | 4 | 4.1 |
| 40 | F2I | 2IQG |  | C32 H38 F2 I N3 O3 | 5 | 677 | 6 | 3 | 6.1 |

Table S2: Screened hits’ compound IDs, SMILES, along with their RMSD values, molecular weight (MW), topological polar surface area (TPSA), and Lipinski Rule of Five.

| **No.** | **Compound ID** | **SMILES** | **RMSD** | **MW** | **TPSA** | **Lipinski Rule of Five** |
| --- | --- | --- | --- | --- | --- | --- |
| 1 | CUM-0095 | c1cc(=O)oc(c12)ccc(c2)Nc(c3)nnn3CCNc4ccn[nH]4 | 0.5 | 337.13 | 116.89 | Accepted |
| 2 | CUM-0119 | c1cc(=O)oc(c12)ccc(c2)Nc(c3)nnn3CO[C@]4(C)CCNC4 | 0.4 | 341.15 | 94.21 | Accepted |
| 3 | CUM-0158 | c1cc(=O)oc(c12)ccc(c2)Nc(c3)nnn3CNc([nH]4)ccc4C | 0.4 | 336.13 | 100.77 | Accepted |
| 4 | CUM-0173 | c1cc(=O)oc(c12)ccc(c2)Nc(c3)nnn3CCOc4ccn[nH]4 | 0.5 | 338.11 | 110.86 | Accepted |
| 5 | CUM-0180 | c1cc(=O)oc(c12)ccc(c2)Nc(c3)nnn3CO[C@H](C4=O)CCN4 | 0.4 | 341.11 | 111.28 | Accepted |
| 6 | CUM-0184 | c1cc(=O)oc(c12)ccc(c2)Nc(c3)nnn3CCOc4ncc[nH]4 | 0.5 | 338.11 | 110.86 | Accepted |
| 7 | CUM-0196 | c1cc(=O)oc(c12)ccc(c2)Nc(c3)nnn3CCc4cc([nH]n4)O | 0.5 | 338.11 | 121.6 | Accepted |
| 8 | CUM-0199 | c1cc(=O)oc(c12)ccc(c2)Nc(c3)nnn3COc([nH]n4)cc4C | 0.3 | 338.11 | 110.86 | Accepted |
| 9 | CUM-0226 | c1cc(=O)oc(c12)ccc(c2)Nc(c3)nnn3CN[C@H](C4(C)C)CN4 | 0.3 | 340.16 | 97.01 | Accepted |
| 10 | CUM-0227 | c1cc(=O)oc(c12)ccc(c2)Nc(c3)nnn3CN4C[C@@H](N)C4(C)C | 0.4 | 340.16 | 102.21 | Accepted |
| 11 | CUM-0234 | c1cc(=O)oc(c12)ccc(c2)Nc(c3)nnn3CCc4c(O)c[nH]n4 | 0.5 | 338.11 | 121.86 | Accepted |
| 12 | CUM-0235 | c1cc(=O)oc(c12)ccc(c2)Nc(c3)nnn3COc(c4C)c[nH]n4 | 0.5 | 338.11 | 110.86 | Accepted |
| 13 | CUM-0256 | c1cc(=O)oc(c12)ccc(c2)Nc(c3)nnn3CN(C)CC4(O)CC4 | 0.5 | 341.15 | 96.42 | Accepted |
| 14 | CUM-0262 | c1cc(=O)oc(c12)ccc(c2)Nc(c3)nnn3CCOCC4(O)CC4 | 0.6 | 342.13 | 102.41 | Accepted |
| 15 | CUM-0284 | c1cc(=O)oc(c12)ccc(c2)Nc(c3)nnn3CO[C@@H](C)C4(N)CC4 | 0.3 | 341.15 | 108.2 | Accepted |
| 16 | CUM-0319 | c1cc(=O)oc(c12)ccc(c2)Nc(c3)nnn3CN(C)C4(CC4)CN | 0.3 | 340.16 | 102.21 | Accepted |
| 17 | CUM-0350 | c1cc(=O)oc(c12)ccc(c2)Nc(c3)nnn3CCc4nc(O)c[nH]4 | 0.6 | 338.11 | 121.86 | Accepted |
| 18 | CUM-0351 | c1cc(=O)oc(c12)ccc(c2)Nc(c3)nnn3COc(c[nH]4)nc4C | 0.5 | 338.11 | 110.86 | Accepted |
| 19 | CUM-0421 | c1cc(=O)oc(c12)ccc(c2)Nc(c3)nnn3CCc4cc(C)[nH]n4 | 0.5 | 336.13 | 101.63 | Accepted |
| 20 | CUM-0469 | c1cc(=O)oc(c12)ccc(c2)Nc(c3)nnn3Cc(o4)ccc4CO | 0.5 | 338.1 | 106.32 | Accepted |
| 21 | CUM-0470 | c1cc(=O)oc(c12)ccc(c2)Nc(c3)nnn3COC[C@@H]4CCCN4 | 0.5 | 341.15 | 94.21 | Accepted |
| 22 | CUM-0507 | c1cc(=O)oc(c12)ccc(c2)Nc(c3)nnn3CNCC4(CO)CC4 | 0.5 | 341.15 | 105.21 | Accepted |
| 23 | CUM-0584 | c1cc(=O)oc(c12)ccc(c2)Nc(c3)nnn3C\N=c(cc4)\[nH]n4C | 0.3 | 337.13 | 106.03 | Accepted |
| 24 | CUM-0603 | c1cc(=O)oc(c12)ccc(c2)Nc(c3)nnn3CO[C@@H](C4(C)C)CN4 | 0.3 | 341.15 | 94.21 | Accepted |
| 25 | CUM-0605 | c1cc(=O)oc(c12)ccc(c2)Nc(c3)nnn3CO[C@@H](CC4=O)CN4 | 0.4 | 341.11 | 111.28 | Accepted |
| 26 | CUM-0620 | c1cc(=O)oc(c12)ccc(c2)Nc(c3)nnn3CCN4C[C@@H](O)CC4 | 0.6 | 341.15 | 96.42 | Accepted |
| 27 | CUM-0692 | c1cc(=O)oc(c12)ccc(c2)Nc(c3)nnn3COC[C@@H](N)C4CC4 | 0.5 | 341.15 | 108.2 | Accepted |
| 28 | CUM-0704 | c1cc(=O)oc(c12)ccc(c2)Nc(c3)nnn3COC4(CC)CNC4 | 0.2 | 341.15 | 94.21 | Accepted |
| 29 | CUM-0715 | c1cc(=O)oc(c12)ccc(c2)Nc(c3)nnn3CCOCC4(N)CC4 | 0.3 | 341.15 | 108.2 | Accepted |
| 30 | CUM-0735 | c1cc(=O)oc(c12)ccc(c2)Nc(c3)nnn3CN[C@]4(C)C[C@@H](C4)O | 0.5 | 341.15 | 105.21 | Accepted |
| 31 | CUM-0736 | c1cc(=O)oc(c12)ccc(c2)Nc(c3)nnn3CO[C@H](C4)C[C@@]4(C)N | 0.5 | 341.15 | 108.2 | Accepted |
| 32 | CUM-0745 | c1cc(=O)oc(c12)ccc(c2)Nc(c3)nnn3CNC4(CC)CNC4 | 0.2 | 340.16 | 97.01 | Accepted |
| 33 | CUM-0764 | c1cc(=O)oc(c12)ccc(c2)Nc(c3)nnn3CCNc4cnc[nH]4 | 0.5 | 337.13 | 113.66 | Accepted |
| 34 | CUM-0797 | c1cc(=O)oc(c12)ccc(c2)Nc(c3)nnn3CO[C@@H](CN)C4CC4 | 0.5 | 341.15 | 108.2 | Accepted |
| 35 | CUM-0882 | c1cc(=O)oc(c12)ccc(c2)Nc(c3)nnn3CCN(C4=O)CCN4 | 0.5 | 340.13 | 105.29 | Accepted |
| 36 | CUM-0932 | c1cc(=O)oc(c12)ccc(c2)Nc(c3)nnn3CCNc4ncc[nH]4 | 0.4 | 337.13 | 116.89 | Accepted |
| 37 | CUM-0965 | c1cc(=O)oc(c12)ccc(c2)Nc(c3)nnn3CNC4(CO)CCC4 | 0.5 | 341.15 | 105.21 | Accepted |
| 38 | CUM-0994 | c1cc(=O)oc(c12)ccc(c2)Nc(c3)nnn3CCN(C4)CC4(C)N | 0.4 | 340.16 | 102.21 | Accepted |
| 39 | CUM-0997 | c1cc(=O)oc(c12)ccc(c2)Nc(c3)nnn3CCN(C4)CC4(C)O | 0.4 | 341.15 | 96.42 | Accepted |
| 40 | CUM-1026 | c1cc(=O)oc(c12)ccc(c2)Nc(c3)nnn3CNc4cc[nH]c4 | 0.6 | 322.12 | 100.77 | Accepted |
| 41 | CUM-1041 | c1cc(=O)oc(c12)ccc(c2)Nc(c3)nnn3COc4ncc[nH]4 | 0.3 | 324.1 | 110.86 | Accepted |
| 42 | CUM-1064 | c1cc(=O)oc(c12)ccc(c2)Nc(c3)nnn3COc4cn[nH]c4 | 0.5 | 324.1 | 110.86 | Accepted |
| 43 | CUM-1082 | c1cc(=O)oc(c12)ccc(c2)Nc(c3)nnn3CCc4nc[nH]n4 | 0.5 | 323.11 | 114.52 | Accepted |
| 44 | CUM-1085 | c1cc(=O)oc(c12)ccc(c2)Nc(c3)nnn3CNc4ccc[nH]4 | 0.4 | 322.12 | 100.77 | Accepted |
| 45 | CUM-1102 | c1cc(=O)oc(c12)ccc(c2)Nc(c3)nnn3COc4c[nH]cn4 | 0.4 | 324.1 | 110.86 | Accepted |
| 46 | CUM-1106 | c1cc(=O)oc(c12)ccc(c2)Nc(c3)nnn3COc4ccc[nH]4 | 0.5 | 323.1 | 97.97 | Accepted |
| 47 | CUM-1175 | c1cc(=O)oc(c12)ccc(c2)Nc(c3)nnn3COC4(CN)CC4 | 0.3 | 327.13 | 108.2 | Accepted |
| 48 | CUM-1226 | c1cc(=O)oc(c12)ccc(c2)Nc(c3)nnn3C[N-][C@@H]4CCNC4 | 0.4 | 326.15 | 97.01 | Accepted |
| 49 | CUM-1227 | c1cc(=O)oc(c12)ccc(c2)Nc(c3)nnn3CN(C4)CC[C@H]4[NH-] | 0.5 | 326.15 | 102.21 | Accepted |
| 50 | CUM-1228 | c1cc(=O)oc(c12)ccc(c2)Nc(c3)nnn3COc4ccn[nH]4 | 0.5 | 324.1 | 110.86 | Accepted |
| 61 | CUM-1236 | c1cc(=O)oc(c12)ccc(c2)Nc(c3)nnn3C/N=C(/O)C4CC4 | 0.3 | 325.12 | 102.05 | Accepted |
| 52 | CUM-1239 | c1cc(=O)oc(c12)ccc(c2)Nc(c3)nnn3COc4cc[nH]c4 | 0.3 | 323.1 | 97.97 | Accepted |
| 53 | CUM-1260 | c1cc(=O)oc(c12)ccc(c2)Nc(c3)nnn3COC4(C)CNC4 | 0.5 | 327.13 | 94.21 | Accepted |
| 54 | CUM-1267 | c1cc(=O)oc(c12)ccc(c2)Nc(c3)nnn3CN(C4)CC[C@@H]4N | 0.2 | 326.15 | 102.21 | Accepted |
| 55 | CUM-1268 | c1cc(=O)oc(c12)ccc(c2)Nc(c3)nnn3CO[C@@H]4CCNC4 | 0.5 | 327.13 | 94.21 | Accepted |
| 56 | CUM-1269 | c1cc(=O)oc(c12)ccc(c2)Nc(c3)nnn3CN(C4)CC[C@H]4O | 0.5 | 327.13 | 96.42 | Accepted |
| 57 | CUM-1305 | c1cc(=O)oc(c12)ccc(c2)Nc(c3)nnn3CNC4(C)CNC4 | 0.4 | 326.15 | 97.01 | Accepted |
| 58 | CUM-1333 | c1cc(=O)oc(c12)ccc(c2)Nc(c3)nnn3CCc4cc[nH]n4 | 0.3 | 322.12 | 101.63 | Accepted |
| 59 | CUM-1389 | c1cc(=O)oc(c12)ccc(c2)Nc(c3)nnn3COC4CNC4 | 0.5 | 313.12 | 94.21 | Accepted |
| 60 | CUM-1390 | c1cc(=O)oc(c12)ccc(c2)Nc(c3)nnn3CN(C4)CC4O | 0.3 | 313.12 | 96.42 | Accepted |
| 61 | CUM-1391 | c1cc(=O)oc(c12)ccc(c2)Nc(c3)nnn3CNC4CNC4 | 0.4 | 312.13 | 97.01 | Accepted |
| 62 | CUM-1392 | c1cc(=O)oc(c12)ccc(c2)Nc(c3)nnn3CN(C4)CC4N | 0.3 | 312.13 | 102.21 | Accepted |
| 63 | CUM-1426 | c1cc(=O)oc(c12)ccc(c2)Nc(c3)nnn3CN[C@H](C)C(C)(C)O | 0.4 | 343.16 | 105.21 | Accepted |
| 64 | CUM-1437 | c1cc(=O)oc(c12)ccc(c2)Nc(c3)nnn3CCN(C)C[C@H](C)O | 0.5 | 343.16 | 96.42 | Accepted |
| 65 | CUM-1438 | c1cc(=O)oc(c12)ccc(c2)Nc(c3)nnn3CCNC(=O)NCC | 0.4 | 342.14 | 114.08 | Accepted |
| 66 | CUM-1447 | c1cc(=O)oc(c12)ccc(c2)Nc(c3)nnn3CN(C)CC(=O)NC | 0.6 | 342.14 | 105.29 | Accepted |
| 67 | CUM-1455 | c1cc(=O)oc(c12)ccc(c2)Nc(c3)nnn3CCNC(=O)[C@H](C)O | 0.5 | 343.13 | 122.28 | Accepted |
| 68 | CUM-1470 | c1cc(=O)oc(c12)ccc(c2)Nc(c3)nnn3CCOC(C)(C)CN | 0.6 | 343.16 | 108.2 | Accepted |
| 69 | CUM-1499 | c1cc(=O)oc(c12)ccc(c2)Nc(c3)nnn3CN[C@@H](C(C)C)CO | 0.6 | 343.16 | 105.21 | Accepted |
| 70 | CUM-1520 | c1cc(=O)oc(c12)ccc(c2)Nc(c3)nnn3CCNC(C)(C)CO | 0.6 | 343.16 | 105.21 | Accepted |
| 71 | CUM-1526 | c1cc(=O)oc(c12)ccc(c2)Nc(c3)nnn3CN[C@@](C)(CO)CC | 0.5 | 343.16 | 105.21 | Accepted |
| 72 | CUM-1536 | c1cc(=O)oc(c12)ccc(c2)Nc(c3)nnn3CCOCC(C)(C)N | 0.6 | 343.16 | 108.2 | Accepted |
| 73 | CUM-1542 | c1cc(=O)oc(c12)ccc(c2)Nc(c3)nnn3CCN(C)C[C@@H](C)N | 0.6 | 342.18 | 102.21 | Accepted |
| 74 | CUM-1545 | c1cc(=O)oc(c12)ccc(c2)Nc(c3)nnn3CCOCC(C)(C)O | 0.6 | 344.15 | 102.41 | Accepted |
| 75 | CUM-1555 | c1cc(=O)oc(c12)ccc(c2)Nc(c3)nnn3CO[C@@H](C(C)C)CN | 0.5 | 343.16 | 108.2 | Accepted |
| 76 | CUM-1557 | c1cc(=O)oc(c12)ccc(c2)Nc(c3)nnn3CNC[C@](C)(O)CC | 0.5 | 343.16 | 105.21 | Accepted |
| 77 | CUM-1568 | c1cc(=O)oc(c12)ccc(c2)Nc(c3)nnn3CCNCC(C)(C)O | 0.4 | 343.16 | 105.21 | Accepted |
| 78 | CUM-1581 | c1cc(=O)oc(c12)ccc(c2)Nc(c3)nnn3CCOCC(=O)NC | 0.6 | 343.13 | 111.28 | Accepted |
| 79 | CUM-1592 | c1cc(=O)oc(c12)ccc(c2)Nc(c3)nnn3CN(C)C(C)(C)CN | 0.5 | 342.18 | 102.21 | Accepted |
| 80 | CUM-1593 | c1cc(=O)oc(c12)ccc(c2)Nc(c3)nnn3CCNC(C)(C)CN | 0.6 | 342.18 | 111 | Accepted |
| 81 | CUM-1601 | c1cc(=O)oc(c12)ccc(c2)Nc(c3)nnn3CC[C@@](C)(N)CNC | 0.6 | 342.18 | 111 | Accepted |
| 82 | CUM-1602 | c1cc(=O)oc(c12)ccc(c2)Nc(c3)nnn3CNC(C)(C)CNC | 0.5 | 342.18 | 97.01 | Accepted |
| 83 | CUM-1604 | c1cc(=O)oc(c12)ccc(c2)Nc(c3)nnn3CCNCC(C)(C)N | 0.5 | 342.18 | 111 | Accepted |
| 84 | CUM-1616 | c1cc(=O)oc(c12)ccc(c2)Nc(c3)nnn3COCC(C)(C)CN | 0.4 | 343.16 | 108.2 | Accepted |
| 85 | CUM-1626 | c1cc(=O)oc(c12)ccc(c2)Nc(c3)nnn3COCC(F)(C)C | 0.4 | 332.13 | 82.18 | Accepted |
| 86 | CUM-1661 | c1cc(=O)oc(c12)ccc(c2)Nc(c3)nnn3CCN[C@H](C)CN | 0.6 | 328.16 | 111 | Accepted |
| 87 | CUM-1676 | c1cc(=O)oc(c12)ccc(c2)Nc(c3)nnn3CCNC[C@@H](C)O | 0.4 | 329.15 | 105.21 | Accepted |
| 88 | CUM-1679 | c1cc(=O)oc(c12)ccc(c2)Nc(c3)nnn3CCNC(=O)CO | 0.6 | 329.11 | 122.28 | Accepted |
| 89 | CUM-1686 | c1cc(=O)oc(c12)ccc(c2)Nc(c3)nnn3CNC(C)(C)CO | 0.5 | 329.15 | 105.21 | Accepted |
| 90 | CUM-1695 | c1cc(=O)oc(c12)ccc(c2)Nc(c3)nnn3CCNC(=O)NC | 0.5 | 328.13 | 114.08 | Accepted |
| 91 | CUM-1746 | c1cc(=O)oc(c12)ccc(c2)Nc(c3)nnn3CCOC[C@H](C)N | 0.3 | 329.15 | 108.2 | Accepted |
| 92 | CUM-0032 | c1cc(=O)oc(c12)ccc(c2)Nc(c3)nnn3CO[C@@]4(C)C[C@H](C4)N | 0.3 | 342.15 | 108.2 | Accepted |

Table S3: RMSD (Å) values for docking validation of ligands in different BACE1 structures, calculated relative to native binding poses.

| **PDB ID** | **Validation Type** | **RMSD (Å)** |
| --- | --- | --- |
| 2VA7 | Redocking | 0.12 |
| 2VA6 | Redocking | 0.15 |
| 2VA5 | Redocking | 0.25 |
| 2OHT | Redocking | 0.10 |

*Table S4: Docking-based virtual screening results of 92 hits with BACE1 (PDB ID: 2VA7).*

| **No.** | **Docked Compounds** | **Binding Energy** **ΔG (kcal/mol)** |
| --- | --- | --- |
| 1 | 2va7_refined_CUM-0199_uff_E=717.05 | -9.2 |
| 2 | 2va7_refined_CUM-0158_uff_E=622.72 | -8.9 |
| 3 | 2va7_refined_CUM-0196_uff_E=652.14 | -8.7 |
| 4 | 2va7_refined_CUM-0351_uff_E=689.58 | -8.7 |
| 5 | 2va7_refined_CUM-0421_uff_E=648.31 | -8.7 |
| 6 | 2va7_refined_CUM-0350_uff_E=643.20 | -8.6 |
| 7 | 2va7_refined_CUM-1228_uff_E=614.02 | -8.6 |
| 8 | 2va7_refined_CUM-0234_uff_E=658.16 | -8.5 |
| 9 | 2va7_refined_CUM-0736_uff_E=989.66 | -8.5 |
| 10 | 2va7_refined_CUM-1333_uff_E=637.59 | -8.5 |
| 11 | 2va7_refined_CUM-0173_uff_E=669.01 | -8.4 |
| 12 | 2va7_refined_CUM-1082_uff_E=623.87 | -8.4 |
| 13 | 2va7_refined_CUM-1604_uff_E=535.92 | -8.4 |
| 14 | 2va7_refined_CUM-1695_uff_E=1068.84 | -8.4 |
| 15 | 2va7_refined_CUM-0095_uff_E=645.59 | -8.3 |
| 16 | 2va7_refined_CUM-0227_uff_E=1023.05 | -8.3 |
| 17 | 2va7_refined_CUM-0584_uff_E=675.04 | -8.3 |
| 18 | 2va7_refined_CUM-0605_uff_E=600.39 | -8.3 |
| 19 | 2va7_refined_CUM-0932_uff_E=634.32 | -8.3 |
| 20 | 2va7_refined_CUM-1085_uff_E=606.08 | -8.3 |
| 21 | 2va7_refined_CUM-1227_uff_E=582.59 | -8.3 |
| 22 | 2va7_refined_CUM-1102_uff_E=607.76 | -8.2 |
| 23 | 2va7_refined_CUM-1102_uff_E=608.49 | -8.2 |
| 24 | 2va7_refined_CUM-1106_uff_E=584.00 | -8.2 |
| 25 | 2va7_refined_CUM-0235_uff_E=641.29 | -8.1 |
| 26 | 2va7_refined_CUM-1041_uff_E=604.69 | -8.1 |
| 27 | 2va7_refined_CUM-1269_uff_E=590.19 | -8.1 |
| 28 | 2va7_refined_CUM-0180_uff_E=612.00 | -8 |
| 29 | 2va7_refined_CUM-1064_uff_E=612.48 | -8 |
| 30 | 2va7_refined_CUM-1268_uff_E=649.27 | -8 |
| 31 | 2va7_refined_CUM-1390_uff_E=937.99 | -8 |
| 32 | 2va7_refined_CUM-1392_uff_E=953.62 | -8 |
| 33 | 2va7_refined_CUM-1438_uff_E=470.36 | -8 |
| 34 | 2va7_refined_CUM-1455_uff_E=1092.64 | -8 |
| 35 | 2va7_refined_CUM-0184_uff_E=669.42 | -7.9 |
| 36 | 2va7_refined_CUM-0882_uff_E=720.94 | -7.9 |
| 37 | 2va7_refined_CUM-1026_uff_E=626.76 | -7.9 |
| 38 | 2va7_refined_CUM-1226_uff_E=625.66 | -7.9 |
| 39 | 2va7_refined_CUM-1236_uff_E=1661.74 | -7.9 |
| 40 | 2va7_refined_CUM-0469_uff_E=604.59 | -7.8 |
| 41 | 2va7_refined_CUM-0620_uff_E=676.47 | -7.8 |
| 42 | 2va7_refined_CUM-0745_uff_E=978.44 | -7.8 |
| 43 | 2va7_refined_CUM-0994_uff_E=996.04 | -7.8 |
| 44 | 2va7_refined_CUM-0997_uff_E=989.78 | -7.8 |
| 45 | 2va7_refined_CUM-1267_uff_E=621.61 | -7.8 |
| 46 | 2va7_refined_CUM-1305_uff_E=938.83 | -7.8 |
| 47 | 2va7_refined_CUM-1581_uff_E=463.78 | -7.8 |
| 48 | 2va7_refined_CUM-1679_uff_E=456.47 | -7.8 |
| 49 | 2va7_refined_CUM-0119_uff_E=594.48 | -7.7 |
| 50 | 2va7_refined_CUM-0603_uff_E=928.13 | -7.7 |
| 61 | 2va7_refined_CUM-1239_uff_E=594.49 | -7.7 |
| 52 | 2va7_refined_CUM-1545_uff_E=488.78 | -7.7 |
| 53 | 2va7_refined_CUM-0965_uff_E=988.31 | -7.6 |
| 54 | 2va7_refined_CUM-1391_uff_E=911.68 | -7.6 |
| 55 | 2va7_refined_CUM-1593_uff_E=499.72 | -7.6 |
| 56 | 2va7_refined_CUM-1602_uff_E=495.46 | -7.6 |
| 57 | 2va7_refined_CUM-0032_uff_E=967.52 | -7.6 |
| 58 | 2va7_refined_CUM-0226_uff_E=956.50 | -7.5 |
| 59 | 2va7_refined_CUM-0284_uff_E=1780.24 | -7.5 |
| 60 | 2va7_refined_CUM-0319_uff_E=1727.04 | -7.5 |
| 61 | 2va7_refined_CUM-0692_uff_E=1703.70 | -7.5 |
| 62 | 2va7_refined_CUM-0704_uff_E=955.72 | -7.5 |
| 63 | 2va7_refined_CUM-0735_uff_E=975.39 | -7.5 |
| 64 | 2va7_refined_CUM-1175_uff_E=1718.28 | -7.5 |
| 65 | 2va7_refined_CUM-1260_uff_E=955.64 | -7.5 |
| 66 | 2va7_refined_CUM-1389_uff_E=911.78 | -7.5 |
| 67 | 2va7_refined_CUM-1536_uff_E=504.93 | -7.5 |
| 68 | 2va7_refined_CUM-1601_uff_E=511.70 | -7.5 |
| 69 | 2va7_refined_CUM-0797_uff_E=1683.87 | -7.4 |
| 70 | 2va7_refined_CUM-1592_uff_E=552.89 | -7.4 |
| 71 | 2va7_refined_CUM-1616_uff_E=471.29 | -7.4 |
| 72 | 2va7_refined_CUM-1686_uff_E=489.01 | -7.4 |
| 73 | 2va7_refined_CUM-1499_uff_E=480.55 | -7.3 |
| 74 | 2va7_refined_CUM-1526_uff_E=522.29 | -7.3 |
| 75 | 2va7_refined_CUM-1555_uff_E=448.96 | -7.3 |
| 76 | 2va7_refined_CUM-1676_uff_E=455.26 | -7.3 |
| 77 | 2va7_refined_CUM-0507_uff_E=1749.94 | -7.2 |
| 78 | 2va7_refined_CUM-1557_uff_E=468.68 | -7.2 |
| 79 | 2va7_refined_CUM-1426_uff_E=485.11 | -7.1 |
| 80 | 2va7_refined_CUM-1447_uff_E=462.05 | -7.1 |
| 81 | 2va7_refined_CUM-1520_uff_E=562.57 | -7.1 |
| 82 | 2va7_refined_CUM-1624_uff_E=521.23 | -7.1 |
| 83 | 2va7_refined_CUM-0262_uff_E=1753.85 | -7 |
| 84 | 2va7_refined_CUM-0470_uff_E=556.11 | -7 |
| 85 | 2va7_refined_CUM-1437_uff_E=546.01 | -7 |
| 86 | 2va7_refined_CUM-0256_uff_E=1741.03 | -6.9 |
| 87 | 2va7_refined_CUM-0715_uff_E=1771.93 | -6.9 |
| 88 | 2va7_refined_CUM-1542_uff_E=513.59 | -6.9 |
| 89 | 2va7_refined_CUM-1661_uff_E=495.39 | -6.9 |
| 90 | 2va7_refined_CUM-1470_uff_E=535.37 | -6.8 |
| 91 | 2va7_refined_CUM-1746_uff_E=493.79 | -6.8 |
| 92 | 2va7_refined_CUM-1568_uff_E=470.28 | -6.7 |
